# Alpha-synuclein dynamics bridge Type-I Interferon response and SARS-CoV-2 replication in peripheral cells

**DOI:** 10.1186/s40659-023-00482-x

**Published:** 2024-01-09

**Authors:** Fiona Limanaqi, Silvia Zecchini, Irma Saulle, Sergio Strizzi, Claudia Vanetti, Micaela Garziano, Gioia Cappelletti, Debora Parolin, Sonia Caccia, Daria Trabattoni, Claudio Fenizia, Mario Clerici, Mara Biasin

**Affiliations:** 1 Department of Biomedical and Clinical Sciences, University of Milan, Via G.B. Grassi, Milan, Italy; 2 Department of Pathophysiology and Transplantation, University of Milan, Via Francesco Sforza, Milan, Italy; 3 IRCCS Fondazione Don Carlo Gnocchi, 20148 Milan, Italy

**Keywords:** Interferon Stimulated Genes (ISG), Alpha-synuclein (SNCA), Alpha-synuclein multimer:monomer ratio, Viral infection, Epithelial lung cells, Endothelial cells, Peripheral blood mononuclear cells (PBMC), PBMC-epithelial cell co-culture

## Abstract

**Background:**

Increasing evidence suggests a double-faceted role of alpha-synuclein (α-syn) following infection by a variety of viruses, including SARS-CoV-2. Although α-syn accumulation is known to contribute to cell toxicity and the development and/or exacerbation of neuropathological manifestations, it is also a key to sustaining anti-viral innate immunity. Consistently with α-syn aggregation as a hallmark of Parkinson's disease, most studies investigating the biological function of α-syn focused on neural cells, while reports on the role of α-syn in periphery are limited, especially in SARS-CoV-2 infection.

**Results:**

Results herein obtained by real time qPCR, immunofluorescence and western blot indicate that α-syn upregulation in peripheral cells occurs as a Type-I Interferon (IFN)-related response against SARS-CoV-2 infection. Noteworthy, this effect mostly involves α-syn multimers, and the dynamic α-syn multimer:monomer ratio. Administration of excess α-syn monomers promoted SARS-CoV-2 replication along with downregulation of IFN-Stimulated Genes (ISGs) in epithelial lung cells, which was associated with reduced α-syn multimers and α-syn multimer:monomer ratio. These effects were prevented by combined administration of IFN-β, which hindered virus replication and upregulated ISGs, meanwhile increasing both α-syn multimers and α-syn multimer:monomer ratio in the absence of cell toxicity. Finally, in endothelial cells displaying abortive SARS-CoV-2 replication, α-syn multimers, and multimer:monomer ratio were not reduced following exposure to the virus and exogenous α-syn, suggesting that only productive viral infection impairs α-syn multimerization and multimer:monomer equilibrium.

**Conclusions:**

Our study provides novel insights into the biology of α-syn, showing that its dynamic conformations are implicated in the innate immune response against SARS-CoV-2 infection in peripheral cells. In particular, our results suggest that promotion of non-toxic α-syn multimers likely occurs as a Type-I IFN-related biological response which partakes in the suppression of viral replication. Further studies are needed to replicate our findings in neuronal cells as well as animal models, and to ascertain the nature of such α-syn conformations.

**Supplementary Information:**

The online version contains supplementary material available at 10.1186/s40659-023-00482-x.

## Background

In the light of the relationship between viral infections and neurological manifestations, several studies focused, in particular, on the role of virus-induced alterations of alpha-synuclein (α-syn), whose aggregation in Lewy bodies defines synucleopathies such as Parkinson’s Disease (PD). This applies to HIV, West Nile virus, Venezuelan equine encephalitis, Influenza (H1N1), and recently, SARS-CoV-2, which have all been documented either to associate with or directly induce α-syn accumulation in the brain [1–12]. However, the mechanisms through which SARS-CoV-2 affects α-syn proteostasis, and the functional significance of such a phenomenon remain unclear. On the one hand, virus-induced α-syn accumulation, as reported for SARS-CoV-2, H1N1, and HIV, would accelerate neuroinflammation, increasing the risk of neurodegeneration, which is in line with the potentially toxic role of α-syn aggregates in neurons [4–12]. On the other hand, α-syn was shown to behave as a native Interferon Stimulated Gene (ISG) and antiviral factor in West Nile virus and Venezuelan equine encephalitis infection [1–3]. Accordingly, protein aggregates may also form as an immune response to infection, building up a local barrier of activated immune-cells while entrapping and neutralizing pathogens [3, 8, 13–16]. This is not surprising when considering the neuro- and immune-modulatory functions of α-syn, which can promote disease pathogenicity or offer protection depending on the cell type and context [17–19].

Interestingly, beyond the brain, α-syn is also expressed, though in lower amounts, in a variety of non-neural human tissues that are potentially susceptible to SARS-CoV-2 infection and/or related sequelae. These encompass the heart, pancreas, skeletal muscles, gut, kidney, adrenal gland, testis, liver, lung, as well as endothelial and immune cells [20–25], and the measurement of α-syn in peripheral immune cells is suggested as promising diagnostic tool for neurodegenerative diseases [17, 26]. The ubiquitous presence of α-syn suggests that it performs important physiological cell functions not restricted to the nervous system.

Even the distribution of different α-syn conformations varies among tissues. As an intrinsically disordered, and highly dynamic protein, α-syn multimers/oligomers beyond monomers might exist in both neuronal and non-neuronal cell populations, including hematopoietic and lung cells [23, 24]. In line with this, a role for α-syn multimers (mostly tetramers), and for the multimer:monomer α-syn ratio in normal cell physiology has been documented, challenging the classic view of high-molecular weight species as mere drivers or neuropathology [24, 27, 28]. For instance, the equilibrium between α-syn multimers and monomers is a key determinant in the fine-tuning of vesicular trafficking. In neuronal synapses, α-syn multimers were shown to slow down endo- and exocytosis, which is instead prevented by α-syn mutations disrupting its multimerization [29]. Again, in endothelial cells, α-syn acts as a negative regulator of Weibel-Palade bodies (WPBs) exocytosis, intracellular granules containing chemokines, adhesion molecules, and inflammatory cytokines [21]. Remarkably, the negative regulation of WPB exocytosis requires the N terminus or the nonamyloid beta-component (NAC) of the Alzheimer disease-associated amyloid region of α-syn, which is known to stabilize higher-order α-syn conformations, suggesting a physiological role of α-syn oligomers/multimers in endothelial cell physiology [21].

Based on these premises, we sought to investigate the role of α-syn in SARS-CoV-2 infection within a variety of cell models including human epithelial lung cell lines (CaLu-3 and A549-hACE2), umbilical vein endothelial cells (HUVECs), and eventually, peripheral blood mononuclear cells (PBMCs).

## Results

### Alpha-synuclein is downregulated at late time-intervals post-SARS-CoV-2 infection in epithelial lung cells

Following pilot experiments validating α-syn mRNA and protein expression within two well-known epithelial lung cell lines that display high susceptibility to SARS-CoV-2 infection (namely, A549-hACE2 and CaLu-3), we sought to investigate whether and how the live virus affects α-syn within these very same cells. The antiviral cytokine IFN-β was also exogenously administered to cells prior to SARS-CoV-2 infection to test potential associations between α-syn expression and Type-I IFNs. Samples were analyzed for synuclein alpha gene (SNCA) mRNA and α-syn protein expression at 24 and 48 h post-infection.

**Fig. 1:**
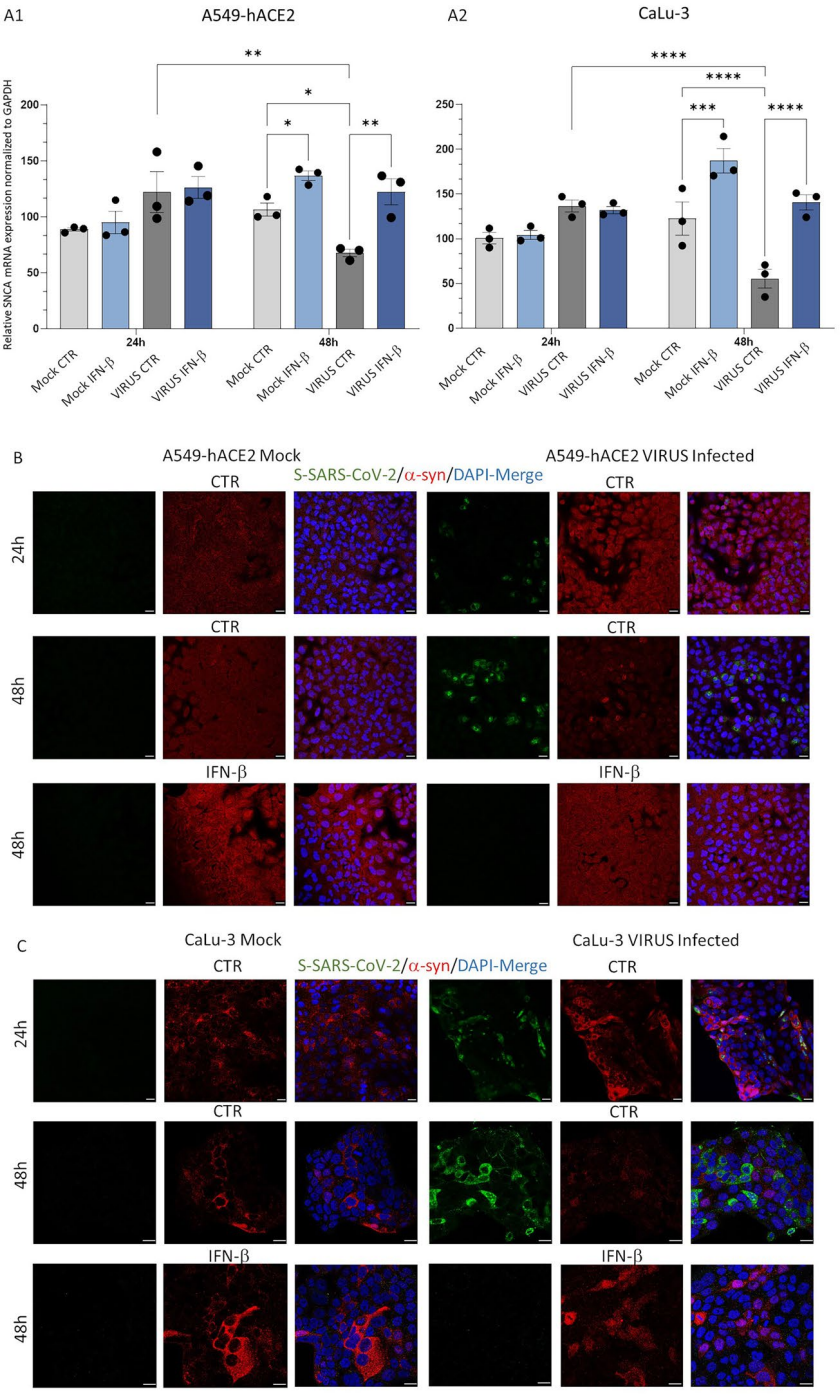
SARS-CoV-2 differently affects SNCA mRNA expression and α-syn protein levels at 24 and 48 h post-infection in A549-hACE2 and CaLu-3 epithelial lung cells. **A** qPCR-based relative quantification of SNCA mRNA in Mock- and SARS-CoV-2-infected cells (MOI 0.05), either in the presence or absence of exogenous IFN-β, 24 and 48 h post-infection. Results are shown as mean ± SEM from n = 3 independent experiments. Statistical analysis was performed by applying Two-way ANOVA, followed by multiple testing correction. To avoid graphs overcrowding, p values are shown for statistically significant groups of interest only. *p < 0.05, **p < 0.01, ***p < 0.001, ****p < 0.0001. **B** and **C** Representative immunofluorescence images for SARS-CoV-2 Spike (S) and α-syn proteins in Mock- and SARS-CoV-2-infected (MOI 0.05) in A549-hACE2 (**B**) and CaLu-3 (**C**) epithelial lung cells 24 and 48 h post-infection, in the absence (CTR) or presence of IFN-β. Cells were fixed in 4% Formaldehyde solution for 15 min and permeabilized with 0.1% of Triton X-100 for 15 min. Images are representative of n = 3 independent experiments. Bars correspond to 20 μm

Transcriptional analysis carried out in both A549-hACE2 and CaLu-3 cells showed that compared with Mock-infected samples, SARS-CoV-2 slightly, but not significantly increased SNCA expression at early time-intervals (24 h) post-infection, which was not modified by IFN-β pretreatment (Fig. 1A). Remarkably, an opposite effect was observed at later time-intervals post-infection. Indeed, at 48 h, a rough 40-to-60% reduction of SNCA mRNA occurred in SARS-CoV-2- compared with Mock-infected cells, which was instead prevented by IFN-β pretreatment (Fig. 1A).

These data were confirmed at protein levels, as shown by combined immunofluorescence for α-syn and SARS-CoV-2 Spike (S) protein in both A549-hACE2 and CaLu-3 cells (Fig. 1B, C, respectively). In detail, at 24 h post-infection, a qualitative increase in α-syn immunostaining was observed in SARS-CoV-2-infected compared to Mock cells, while an opposite scenario occurred at 48 h post-infection. Remarkably, IFN-β pretreatment abolished SARS-CoV-2 replication while rescuing α-syn immunostaining in both cell lines (Fig. 1B, C). Similar to the effects of SARS-CoV-2 at 24 h post-infection, IFN-β promoted α-syn accumulation especially at nuclear level. This seems in line with recent data showing that α-syn-mediated antiviral effects are bound to its nuclear co-localization with STAT2, as confirmed following Type-I IFN stimulation in neurons [2].

### Silencing SNCA by siRNA marginally affects SARS-CoV-2 replication in epithelial lung cells

To investigate whether constitutive α-syn expression plays a role in SARS-CoV-2 infection of epithelial lung cell lines, RNA interference was carried out to silence SNCA in A549-hACE2 cells. No significant alterations in cell viability were detected following transfection with SNCA- compared with non-targeting (NT)-siRNA (Additional file 1: Fig. S1A).

**Fig. 2:**
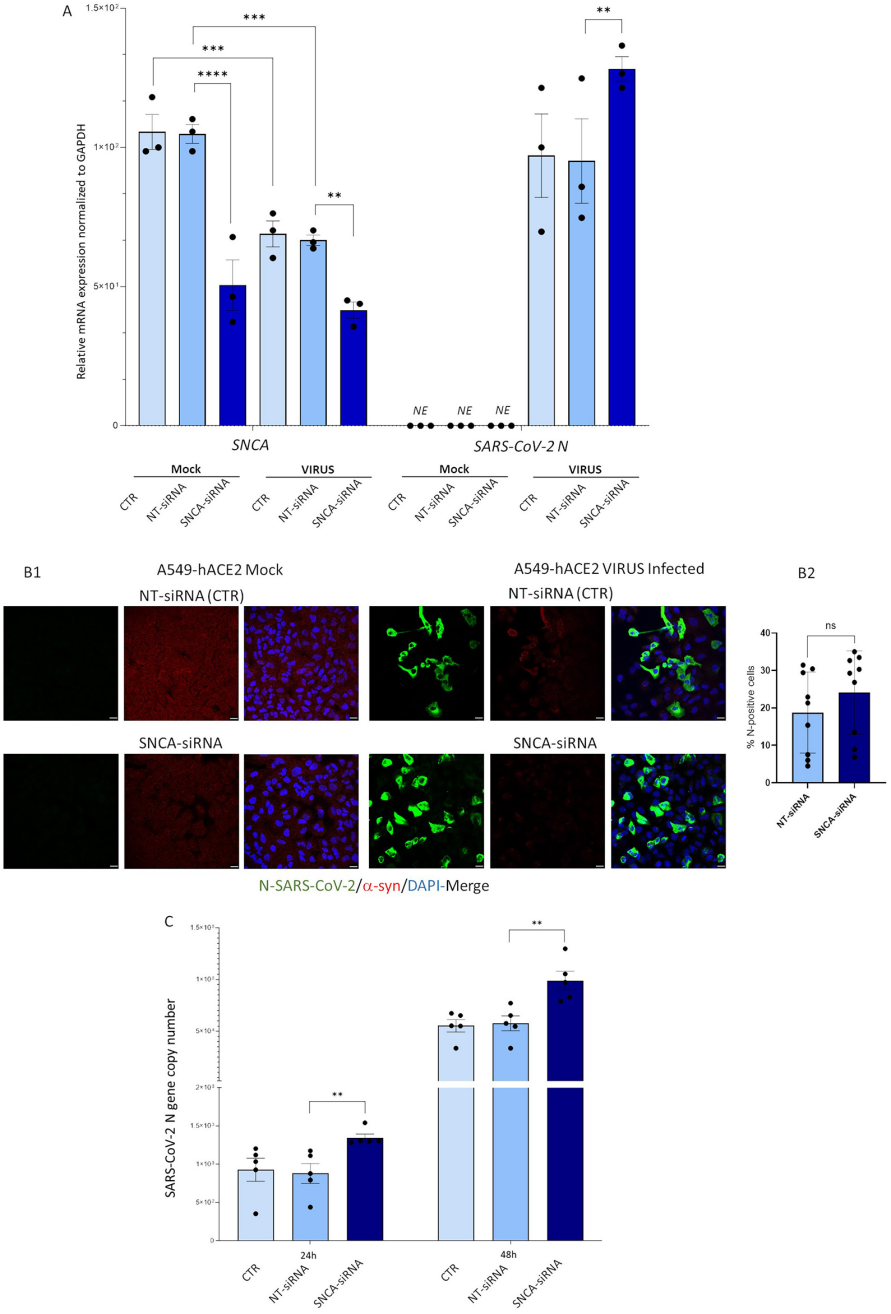
Effects of SNCA silencing in SARS-CoV-2 replication in A549-hACE2 epithelial lung cells. **A** Transcriptional analysis assessing the efficacy of SNCA silencing within A549-hACE2 cells, and related effects in SARS-CoV-2 infection (MOI 0.05) through assessment of intracellular N gene expression. Results are shown as mean ± SEM from n = 3 independent experiments. Values for SNCA and SARS-CoV-2 N mRNA were analyzed by applying One-Way ANOVA followed by multiple testing correction. **p < 0.01, ***p < 0.001, ****p < 0.0001. NE = not expressed. **B1** Representative immunofluorescence experiments showing SNCA-siRNA efficacy at protein levels, and related effects in SARS-CoV-2 infection (MOI 0.05) through intracellular N protein staining. Cells were fixed in 4% Formaldehyde solution for 15 min and permeabilized with 0.1% of Triton X-100 for 15 min. Bars correspond to 20 μm. **B2** Percentage of N-positive cells ± SD calculated as the number of N-positive cells/total cells per microscopic field, from n = 9 microscopic fields per experimental group, from n = 3 independent experiments. Data were analyzed by applying the Student’s *t-test* for comparison between NT-siRNA and SNCA-siRNA groups. ns = non-significant. **C** Quantification of viral replication from cell supernatants through RT-qPCR for the SARS-CoV-2 N gene at 24 and 48 h post-infection in A549-hACE2 cells treated with SNCA-siRNA compared with NT-siRNA. Results are shown as mean ± SEM from n = 5 independent experiments. Values for 24 and 48 h post-infection were analyzed by applying One-way ANOVA. **p < 0.01, ***p < 0.001

Compared with NT-siRNA, SNCA-siRNA produced a rough 50% reduction in SNCA mRNA in Mock cells, and a 35% reduction in SARS-CoV-2-infected cells (Fig. 2A). At intracellular level, a very same 35% increase of the SARS-CoV-2 N gene mRNA was observed in SNCA-siRNA compared with NT-siRNA (Fig. 2A). However, no significant differences in SNCA expression were detected between the SNCA-siRNA Mock *vs.* SNCA-siRNA Virus groups. A marginal, and non-statistically significant, increase in the number of SARS-CoV-2-infected cells was detected in SNCA-siRNA compared with NT-siRNA, as from immunofluorescence experiments (Fig. 2B). Qualitatively, at protein level, and similar to what observed at mRNA, the silencing effect of SNCA-siRNA appeared less pronounced in SARS-CoV-2-infected compared with Mock cells. In fact, the virus per se already produced an evident decrease in both SNCA mRNA (Fig. 2A) and α-syn immunostaining (Fig. 2B). Finally, RT-qPCR-based quantification of extracellular viral titers from cell supernatants showed a weak, though statistically significant increase in SARS-CoV-2 replication in SNCA-siRNA cells compared with NT-siRNA controls, which persisted at 48 h post-infection (Fig. 2C). These data suggest that SNCA downregulation within epithelial lung cells marginally affects SARS-CoV-2 replication.

### Exogenous α-syn monomers, and IFN-β oppositely modulate SARS-CoV-2 infection and formation of permeabilization-resistant α-syn species in infected epithelial lung cells

Based on the previously established capability of exogenously administered extracellular α-syn to be internalized through either passive diffusion or endocytosis [30], we next sought to test whether and how SARS-CoV-2 infection is modulated by a condition of excess α-syn. Thus, exogenous recombinant α-syn monomers (1 μM) were pre-administered to cells both alone and in combination with IFN-β (500 IU/ml) before SARS-CoV-2 infection. Cell viability was not significantly modified by either compound and compared with Mock- and SARS-CoV-2-infected control (CTR) cells, indicating a lack of toxicity in either cell line (Additional file 1: Fig. S1B, S1C).

**Fig. 3:**
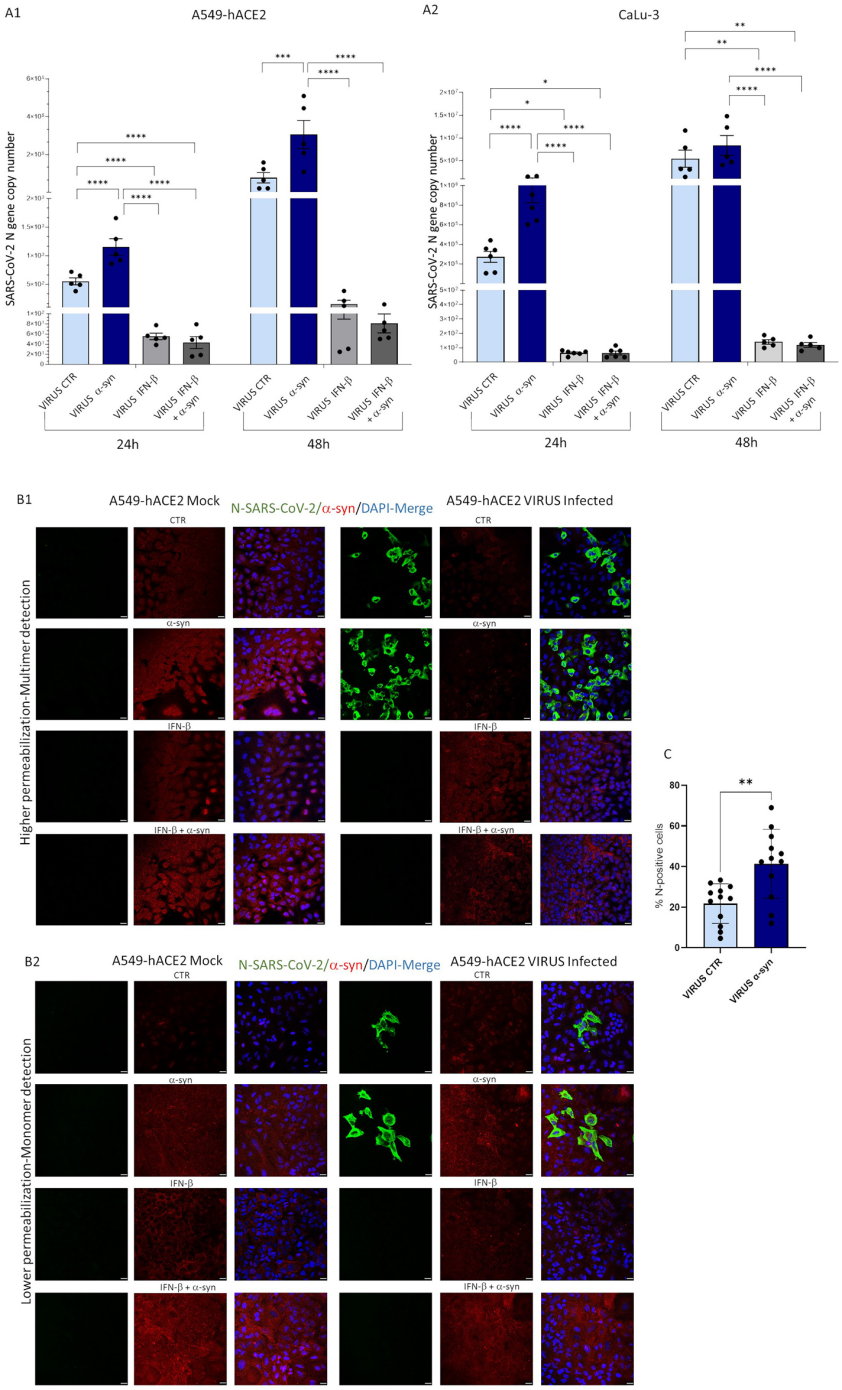
**A1** and **A2** Exogenous administration of α-syn monomers increases SARS-CoV-2 replication in A549-hACE2 and CaLu-3 epithelial cells, which is prevented by IFN-β. Quantification of viral replication from cell supernatants through RT-qPCR for the SARS-CoV-2 N gene in infected A549-hACE2 (**A1**) and CaLu-3 cells (**A2**), 24 and 48 h post-infection (MOI 0.05). **A1** In A549-hACE2 cells, exogenous α-syn monomers significantly increased viral replication compared with infected CTR both at 24 and 48 h post-infection. **A2** In CaLu-3 cells the pro-viral effect of exogenous α-syn monomers was maximally detected at 24 h, while it declined at 48 h post-infection. In both cases, IFN-β, either alone or in combination with exogenous α-syn, completely prevented SARS-CoV-2 replication. Results show SARS-CoV-2 N gene copy numbers quantified from the RNA isolated from 100 μl of cell supernatants. Results are shown as mean ± SEM from n ≥ 5 independent experiments. Values for 24 and 48 h post-infection were analyzed by applying One-Way ANOVA followed by multiple testing correction. *p < 0.05, **p < 0.01, ***p < 0.001, ****p < 0.0001. **B1** and **B2** Representative immunofluorescence images of SARS-CoV-2 Nucleocapsid (N) and α-syn proteins in Mock- and SARS-CoV-2-Infected A549-hACE2 epithelial lung cells 48 h post-infection (MOI 0.05), in the absence and presence of IFN-β, exogenous α-syn monomers, or both. SARS-CoV-2 replication is enhanced in the presence of exogenous α-syn monomers, which impair accumulation of permeabilization-resistant α-syn species that are instead increased by IFN-β. **B1** For visualization of large, permeabilization-resistant α-syn species, that potentially correspond to multimers or intracellular membrane-bound species, cells were fixed for 15 min in 4% Formaldehyde solution and permeabilized with 0.3% of Triton X-100 for 15 min. Bars correspond to 20 μm. **B2** For better preservation, and visualization of small, highly-soluble α-syn species potentially corresponding to monomers, cells were fixed for 15 min in 4% Paraformaldehyde (PFA) and permeabilized with 0.1% of Triton X-100 for 10 min. Bars correspond to 20 μm. Images are representative of n = 3 independent experiments. **C** Quantification of SARS-CoV-2-N-positive cells in SARS-CoV-2-infected CTR vs α-syn-treated SARS-CoV-2-infected cells. Values are expressed as the percentage of N-positive cells ± SD calculated as the number of N-positive cells/total cells per microscopic field from n = 12 microscopic fields per experimental group, from n = 3 independent experiments. Data were analyzed by applying the Student’s *t-test* for comparison between VIRUS CTR and VIRUS α-syn groups. **p < 0.01

Remarkably, qPCR results showed that exogenous α-syn monomers dramatically increase SARS-CoV-2 replication, while pre-treatment with IFN-β hinders viral replication both in the absence and presence of exogenous α-syn (Fig. 3A1, A2). In A549-hACE2 cells, exogenous α-syn monomers led to a two-fold increase in extracellular viral mRNA at 24 h post-infection, which persisted, and was even magnified at 48 h post-infection reaching a three-fold increase compared with untreated infected cells (Fig. 3A1). Within CaLu-3 cells, the pro-viral effect of exogenous α-syn monomers was instead observed to peak 24 h post-infection, with a three-fold increase in extracellular SARS-CoV-2 mRNA compared with untreated cells; however, a non-significant 60% increase compared with untreated infected cells was observed at 48 h post-infection (Fig. 3A2). We speculate that this might be due to the high susceptibility of CaLu-3 cells to SARS-CoV-2, reaching a plateau already at 48 h post-infection. Indeed, at baseline, viral titers in CaLu-3 cells far exceed those detected in A549-heACE2 cells at 24 or 48 h post-infection. In both cell lines, and at both time-points post-infection, pretreatment with IFN-β was able to fully prevent the pro-viral effect of exogenous α-syn monomers (Fig. 3A1, A2). These results were somehow unexpected when considering the increase of α-syn following administration of the antiviral cytokine IFN-β. This prompted us to investigate whether the pro- and anti-viral effects of exogenous α-syn and IFN-β, respectively, are associated with altered dynamics/conformations of internalized α-syn (i.e. accumulation/aggregation).

To such an aim, immunofluorescence experiments were performed with two different cell fixation and permeabilization procedures, which were modified from [31] for better detection of low- vs high-weight α-syn molecular species (monomers, and multimers, respectively), as specified in the Figure legends (Fig. 3B1, B2). Remarkably, an evident qualitative increase in α-syn immunostaining following exogenous α-syn addition, and following both fixation procedures was observed in Mock cells only (Fig. 3B1, B2). Instead, in the presence of SARS-CoV-2, α-syn immunostaining following exogenous α-syn addition was not increased in highly-permeabilized cells (Fig. 3B1), suggesting that the virus hinders formation of permeabilization-resistant α-syn species potentially corresponding to multimers. This effect was associated with an increase in the number of SARS-CoV-2-infected cells compared with untreated infected CTR (Fig. 3C). Again, such an effect was prevented by IFN-β which fully hampered SARS-CoV-2 replication even in the presence of exogenous α-syn (Fig. 3B1). In detail, both when administered alone, and in combination with exogenous α-syn, IFN-β increased permeabilization-resistant multimeric α-syn species in both Mock- and SARS-CoV-2 infected cells compared with CTR (Fig. 3B1). Instead, α-syn monomer staining, as observed through the low-permeabilization procedure, was not significantly affected by IFN-β alone in SARS-CoV-2-infected cells*,* but neither was it decreased when IFN-β was combined with exogenous α-syn (Fig. 3B2). This suggests that, rather than downregulation of α-syn monomers, the promotion of α-syn multimerization counterbalancing the accumulation of monomeric internalized α-syn is associated with the antiviral effects of IFN-β. Thus, in the presence of excess α-syn monomers, as mimicked here through internalization of exogenous recombinant α-syn, SARS-CoV-2 disrupts the dynamic equilibrium between α-syn conformers by impairing the assembly of α-syn monomers into higher-order species.

The cellular mechanisms through which α-syn multimers might partake in the effect of Type-I IFN against SARS-CoV-2 replication were not addressed herein. Coupled with previous evidence indicating a role of α-syn multimers in slowing down vesicle recycling and exocytosis [29], it is possible that the virus downregulates α-syn expression and the assembly of α-syn monomers into higher order conformers for the sake of its replication within intracellular vesicles and the spread from cell-to-cell. Again, the presence of α-syn in the nucleus indicates that it might modulate transcription factors and genes involved in the innate immune response, which remains to be addressed.

The nature of α-syn multimers/oligomers remains controversial, as both physiological, non-toxic, and toxic multimeric/oligomeric species have been described in the literature [19, 24, 32]. To test whether permeabilization-resistant α-syn species likely representing multimers do correspond to beta-sheet rich, amyloid species, Thioflavin-S (Th-S) staining was carried out in Mock- and SARS-CoV-2-infected CaLu-3 cells both in the absence and presence of exogenous α-syn and IFN-β.

Results showed that, similar to what observed in A549-hACE2 cells, SARS-CoV-2 significantly impairs α-syn multimer formation in the presence of exogenous α-syn monomers compared with untreated cells; this effect was once again reversed by IFN-β (Additional file 1: Fig. S2). However, despite a diffuse merging signal which was more pronounced in SARS-CoV-2-infected and IFN-β-pretreated cells, we could not confirm the occurrence of potential amyloid species, as no clear-cut co-localizing puncta were observed in α-syn and Th-S immunostaining in either experimental conditions (Additional file 1: Fig. S2).

**Fig. 4:**
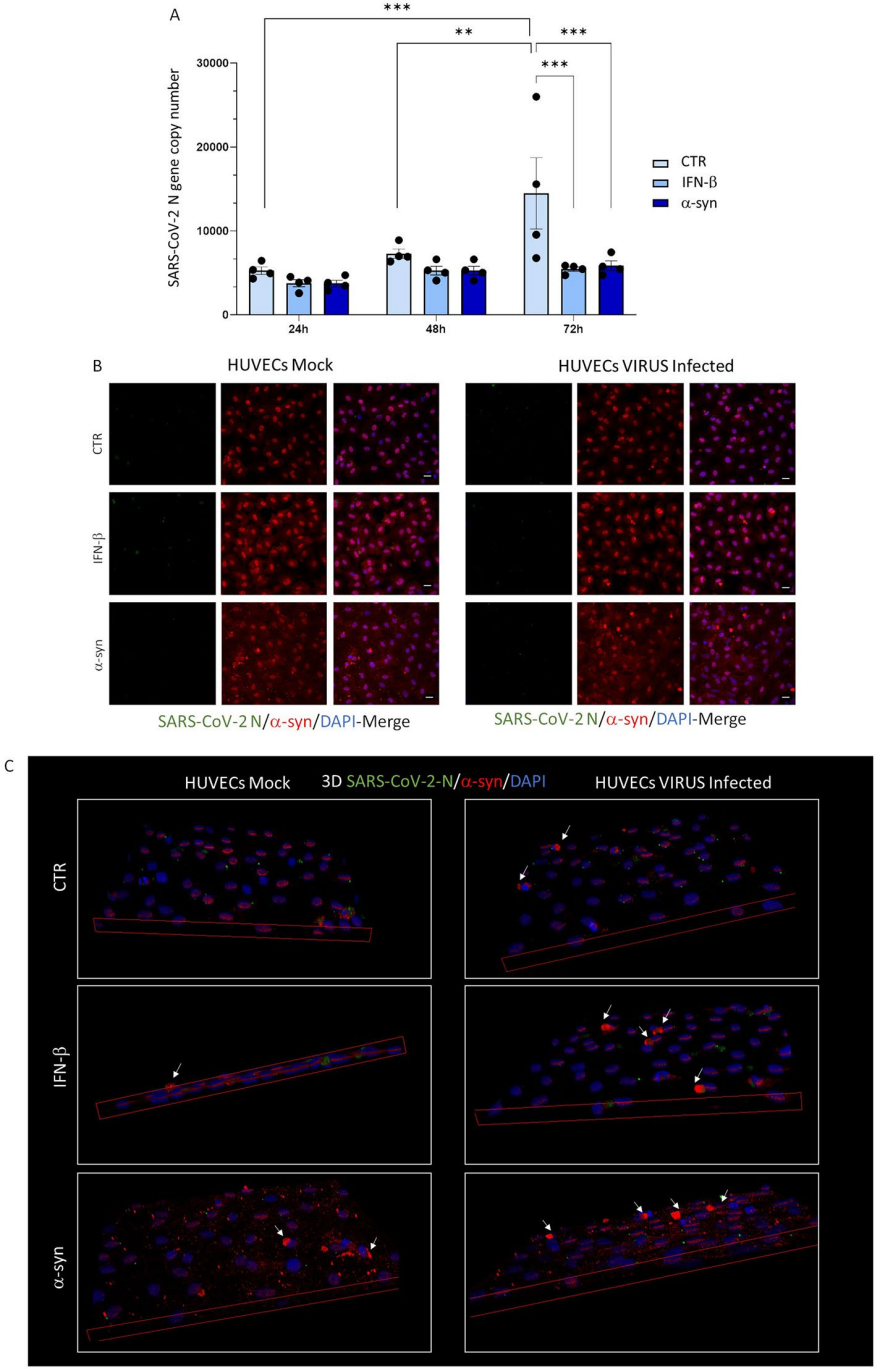
In HUVECS, absence of productive SARS-CoV-2 infection is associated with α-syn accumulation. **A** Quantification of SARS-CoV-2 replication in HUVECs in the absence and presence of α-syn and IFN-β (MOI 1). Results are shown as mean ± SEM from n = 4 independent experiments. Data were analyzed by applying Two-Way ANOVA. **p < 0.01, ***p < 0.001. **B** In the absence of productive viral replication, overall α-syn immunostaining in HUVECs is not decreased by either SARS-CoV-2 infection or the addition of exogenous α-syn monomers. Representative immunofluorescence images for SARS-CoV-2-Nucleocapsid (N), and α-syn in Mock- and SARS-CoV-2-Infected HUVECs 72 h post-infection (MOI 1), in the absence and presence of IFN-β, or exogenous α-syn monomers. Cells were fixed for 15 min in Formaldehyde solution, followed by 15 min permeabilization with 0.1% Triton X-100. Bars correspond to 20 μm. **C** In HUVECs, absence of productive SARS-CoV-2 infection is associated with the occurrence of juxtanuclear α-syn foci. 3D reconstruction of immunofluorescence images for SARS-CoV-2-Nucleocapsid (N), and α-syn proteins in Mock- and SARS-CoV-2-Infected HUVECs 72 h post-infection (MOI 1) shows the presence of juxtanuclear α-syn foci (arrows) which are induced by SARS-CoV-2 and potentiated by IFN-β and exogenous α-syn monomers

Next, we sought to investigate whether the impaired α-syn multimerization observed in virus-susceptible epithelial cells occurs also in experimental models featuring non-productive SARS-CoV-2 infection. To such an aim, human endothelial umbilical vein cells (HUVECs) were used, as they have been widely documented to express very low levels of the SARS‐CoV‐2 receptor ACE2 and the protease TMPRSS2, which restrains their capacity for productive viral infection [33–36]. Initial results showed a time-related increase in SARS-CoV-2 N gene mRNA expression in HUVECs supernatants (Fig. 4A). Administration of either exogenous α-syn or IFN-β led to a 40-to-60% decrease in the amount of detectable extracellular viral RNA, which was statistically significant at 72 h post-infection (Fig. 4A). However, in line with previous evidence [33–36], in all the experimental conditions we failed to detect viral proteins at intracellular level (Fig. 4B). This indicates that HUVECs are not permissive to productive SARS-CoV-2 replication, and that the viral RNA detected in the cell supernatants might be extruded, untranslated viral material. A certain degree of non-specific staining in the green spectrum was constantly detected in HUVECs, as reported by previous studies [37, 38] and shown here both in Mock cells of Fig. 4B, C, and in the immunofluorescence negative control (Additional file 1: Fig. S3A). Contrarily to what observed in epithelial lung cells, in HUVECs, neither SARS-CoV-2 infection per se, nor exogenous α-syn monomer administration resulted in reductions of α-syn immunostaining. Rather, exogenous α-syn administration, similar to IFN-β, enhanced α-syn immunostaining in both Mock and SARS-CoV-2-infected cells compared with untreated controls (Fig. 4B). Remarkably, 3D reconstruction of α-syn immunostaining within HUVECs showed the presence of large, juxtanuclear α-syn foci, which were not observed in epithelial cells, and were mostly evident in SARS-CoV-2-infected HUVECs (Fig. 4C). In fact, while these α-syn foci (arrows) were mostly absent in the Mock CTR group, they became progressively more evident both in IFN-β, and α-syn-treated Mock cells, and mostly in SARS-CoV-2 infected cells, both in CTR, and upon IFN-β- and α-syn addition. Coupled with the evidence of non-productive infection within HUVECs, these data suggest that these large α-syn foci may be part of an innate immune mechanism which is finalized at restraining viral assembly/propagation. Overall, these data support the hypothesis that SARS-CoV-2-induced reduction of α-syn multimer formation observed within epithelial cells but not in HUVECs is ascribable to viral proteins rather than RNA, meanwhile suggesting a possible association between the induction of large α-syn species and restrained viral replication and/or productivity, which remains to be confirmed.

### SARS-CoV-2 reduces multimerization of exogenously added α-syn monomers, which is reversed by IFN-β

**Fig. 5:**
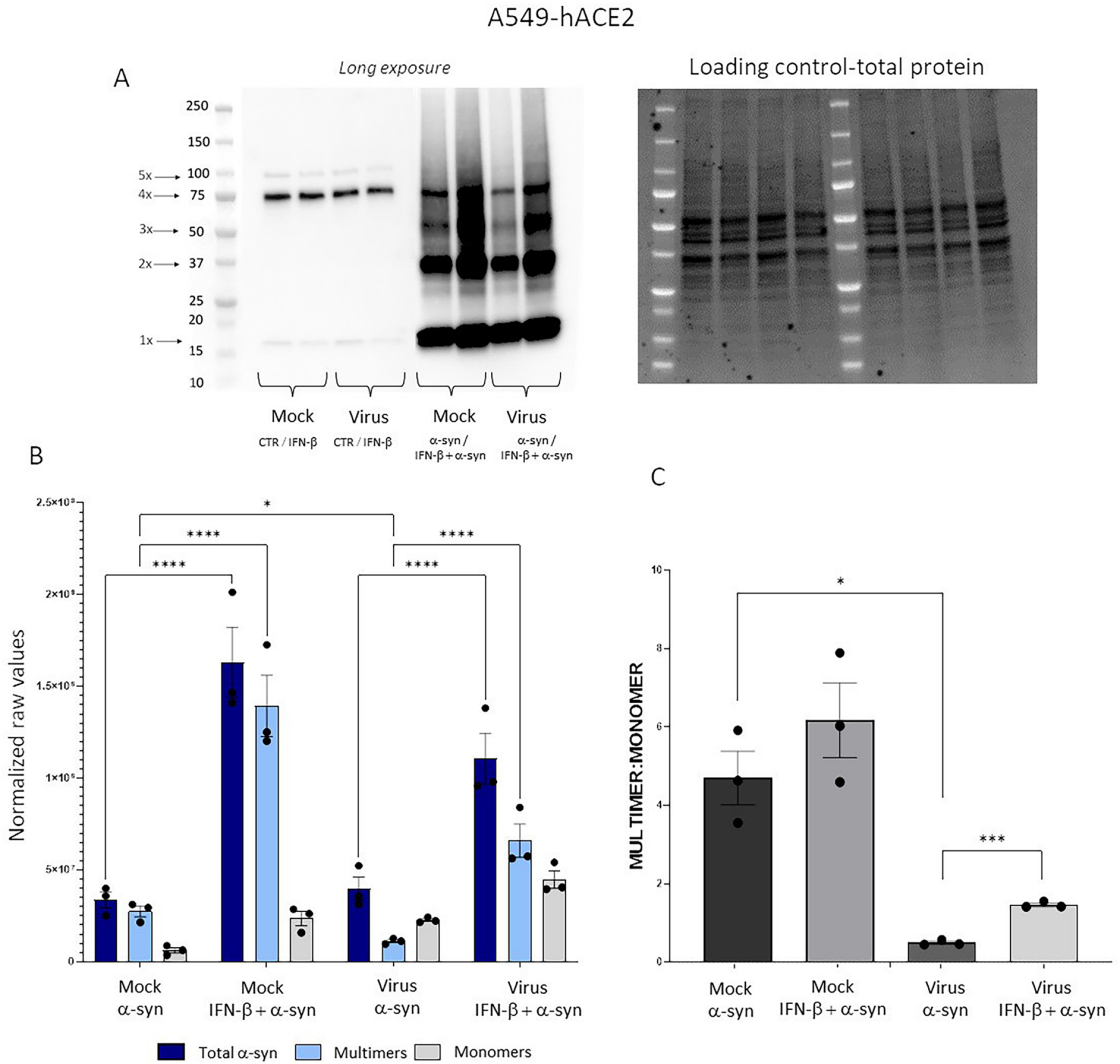
SARS-CoV-2 synergizes with an excess of exogenously added α-syn monomers to reduce α-syn multimer:monomer ratio, which is prevented by IFN-β. **A** Representative western blot, and **B** quantification of total α-syn, α-syn monomers, α-syn multimers, and **C** α-syn multimer:monomer ratio in A549-hACE2 cells 48 h post- Mock and SARS-CoV-2 infection in the presence of exogenously added α-syn. Quantification was performed by normalizing to total protein (Loading control). Results show raw normalized values presented as mean ± SEM from n = 3 independent experiments. Multimer:monomer ratio is expressed as absolute value calculated as “multimers/monomers”. Data were analyzed by applying Two-Way ANOVA (for total, multimer, and monomer α-syn quantification) or One Way ANOVA (for multimer:monomer ratio). *p < 0.05, ***p < 0.001, ****p < 0.0001

To verify if alterations in α-syn multimer:monomer ratio are implicated in the pro-viral effects of exogenously administered α-syn monomers, western blot experiments were carried out in A549-hACE2 epithelial cells, and HUVECs, in all the above-mentioned experimental conditions. Remarkably, in both models, we could detect at baseline both α-syn monomer and multimeric bands migrating approximately at 19 and 75 kDa, respectively (Fig. 5A, Additional file 1: Fig. S4). The latter likely correspond to α-syn tetrameric species/related conformers (19 kDa × 4), which is in line with previous evidence on the physiological occurrence of α-syn multimers in several cell populations [23, 24]. However, detection of endogenous α-syn species at baseline was challenging, as they could be visualized only following long exposures, limiting feasible quantification (Fig. 5A). Thus, only experimental groups with exogenous α-syn were quantified and graphed. When exogenous α-syn was administered to cells, multiple bands corresponding to monomers, dimers, tetramers, and pentamers were detected (Fig. 5A), even at very low exposure (Additional file 1: Fig. S4). This suggests that the observed α-syn signal corresponds in largest part to the exogenously added protein monomers that are internalized and self-assemble into higher-order conformations. Consistently with qPCR and immunofluorescence data, cells with exogenous α-syn displayed an evident increase for SARS-CoV-2 N protein immune-positivity compared with untreated infected cells (Additional file 1: Fig. S4). At first glance, this might support the conclusion that increased α-syn, in either conformation, facilitates SARS-CoV-2 replication. However, the opposite effects of SARS-CoV-2 and IFN-β upon α-syn multimerization, and multimer:monomer ratio argue against such a hypothesis. Indeed SARS-CoV-2 significantly decreased both α-syn multimers (Fig. 5B), and α-syn multimer:monomer ratio (Fig. 5C) compared to Mock cells, with α-syn multimer:monomer ratio dropping from > 4 of Mock to < 1 in the presence of SARS-CoV-2 (Fig. 5C). Total α-syn levels were not affected by SARS-CoV-2, which is conceivable, as the observed signal corresponds in largest part to the exogenously administered, internalized α-syn. Remarkably, SARS-CoV-2-induced reduction of α-syn multimerization was reversed by IFN-β, which significantly increased total α-syn mostly by promoting multimeric species (Fig. 5B) to rescue α-syn multimer:monomer ratio in SARS-CoV-2-infected cells (Fig. 5C). These data suggest that the antiviral effect of IFN-β is associated both with stimulation of endogenous α-syn synthesis, and promotion of α-syn multimerization to counterbalance an excess of α-syn monomers. Again, these data suggest that SARS-CoV-2 synergizes with an excess of α-syn monomers to impair their multimerization, consequently decreasing α-syn multimer:monomer ratio. Thus, rather than changes in the net amount of total α-syn, or single α-syn conformations, alterations in the dynamic α-syn multimer:monomer equilibrium configure as the potential culprit of increased SARS-CoV-2 replication following exogenous α-syn monomer administration.

To summarize, these results indicate that α-syn assembly into multimeric species is neither associated with, nor it represents a culprit of SARS-CoV-2 replication and spread; rather, formation of α-syn multimers is associated with an antiviral, Type-I IFN-related mechanism which is able to contain SARS-CoV-2 replication. This is in part supported by WB data obtained within HUVECs. In fact, in the absence of productive SARS-CoV-2 infection, exogenous α-syn did not significantly decrease α-syn multimers, or α-syn multimer:monomer ratio compared with Mock cells (Additional file 1: Fig. S5). These data strengthen the hypothesis that only productive infection is associated with impaired α-syn multimerization. Thus, an interaction between SARS-CoV-2 proteins and α-syn monomers might be required to alter α-syn multimerization for successful viral replication. Yet, the mechanisms through which Type-I-IFN-induced α-syn multimers, and upregulation of multimer:monomer ratio might contribute to limiting SARS-CoV-2 spread, remain to be investigated.

### SARS-CoV-2-related alterations of α-syn dynamics are associated with mRNA expression of SNCA, interferon stimulated genes, and amplification of pro-inflammatory pathways

**Fig. 6:**
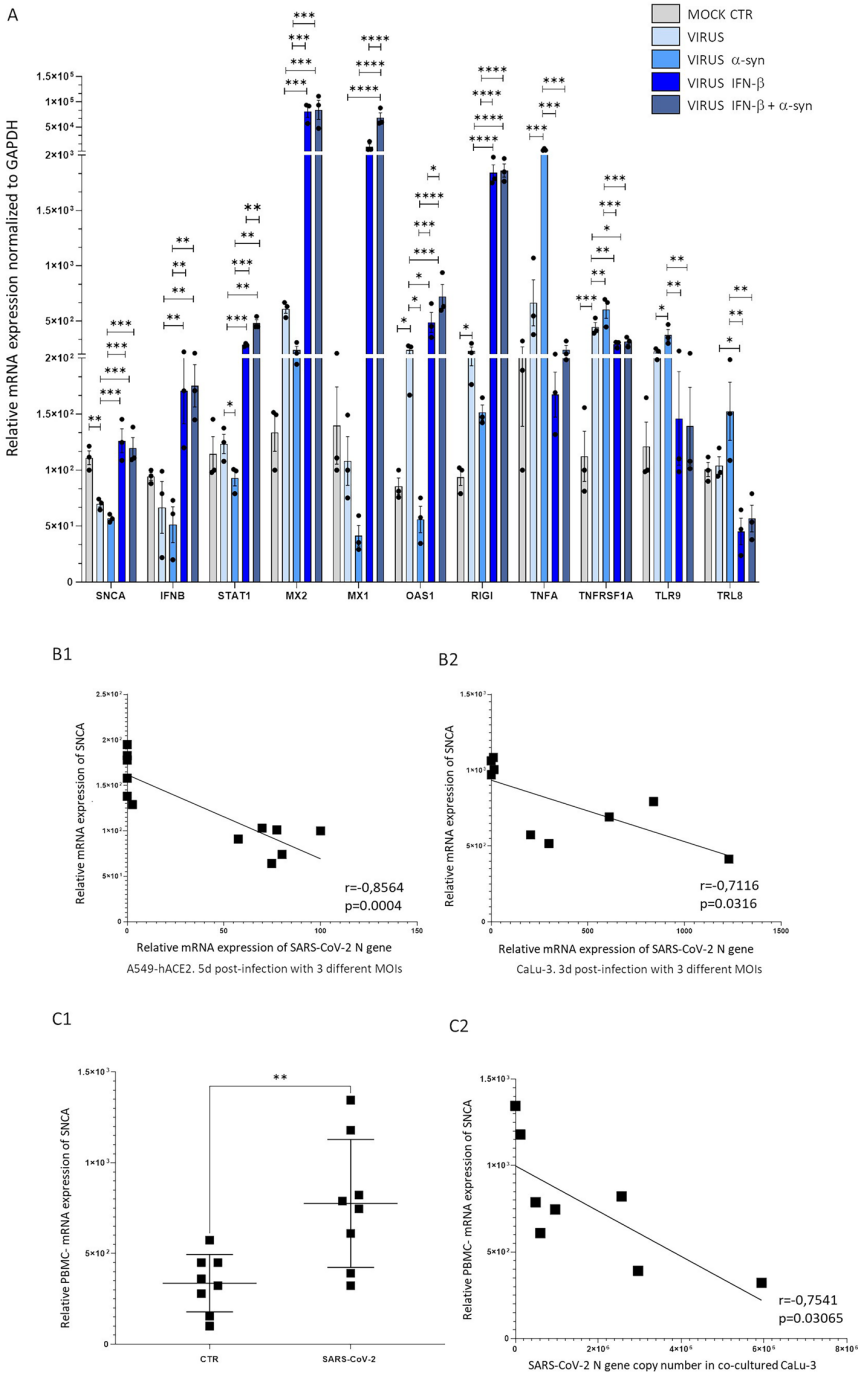
**A** Gene expression changes associated with the pro-viral and anti-viral effects of exogenous α-syn monomers and IFN-β, respectively**.** qPCR-based transcriptional analyses were performed in A549-hACE2 cells 48 h post-infection. Results are expressed as mean ± SEM from n = 3 independent experiments. Data for each gene were analyzed by One Way ANOVA. *p < 0.05, **p < 0.01, ***p < 0.001, ****p < 0.0001. SNCA synclein alpha; IFNB Interferon beta; STAT1 Signal transducer and activator of transcription 1; MX1 Myxovirus Resistance Protein 1; MX2 Myxovirus Resistance Protein 2; OAS1 2'-5'-oligoadenylate synthetase 1, RIG-I retinoic acid-inducible gene I; TNFA tumor necrosis factor alpha, TNFRSF1A tumor necrosis factor receptor superfamily 1A, TLR8 Toll-like receptor 8; Toll-like receptor 9 (TLR9). **B** SNCA mRNA expression is negatively correlated with viral titers at 3 and 5d post-infection of epithelial lung cells**.** The analyses were performed in CaLu-3 and A549-hACE2 cells at 3 and 5d post-infection with SARS-CoV-2 at 3 different MOI (0.01, 0.005, 0.001). Correlation between relative SNCA mRNA expression and SARS-CoV-2 N gene mRNA expression in A549-hACE2 and CaLu-3 cells was calculated through the Pearson’s r coefficient. Statistical significance was calculated through the two-tailed, paired *Student’s t test*. n = 12 for A549-hACE2 cells; n = 9 for CaLu3 cells. **C1** and **C2** SNCA mRNA expression is increased in human PBMCs stimulated with SARS-CoV-2 and is negatively correlated with SARS-CoV-2 replication in co-cultured CaLu-3 cells. **C1** qPCR-based quantification of SNCA mRNA expression in in vitro SARS-CoV-2-exposed human PBMCs compared with Mock CTR. Statistical significance was calculated through the two-tailed, paired *Student’s t test*. The graph shows individual values (n = 8) with mean ± SD. ** p < 0.01. **C2** Correlation analysis between relative SNCA mRNA expression within SARS-CoV-2-exposed PBMCs, and SARS-CoV-2 replication (N gene copy number) in co-cultured CaLu-3 epithelial cells. Correlation was calculated through the Pearson’s r coefficient. Statistical significance was calculated through the two-tailed, paired Student’s t test. n = 8

To investigate potential targets associated with SARS-CoV-2-related alterations of α-syn dynamics, we performed transcriptional analysis of genes associated with innate immune pathways, including SNCA, Type-I IFNs, and Interferon Stimulated Genes (ISGs). Downregulation of both endogenous SNCA and ISGs, along with upregulation of the pro-inflammatory TNFA/TNFR axis and of Toll-like Receptors (TLRs) 8 and 9 was detected in SARS-CoV-2-infected cells that were exogenously administered α-syn monomers compared with untreated infected ones. These effects were fully reversed by IFN-β (Fig. 6A). Interestingly, upregulation of specific ISGs, such as STAT1, MX1, and OAS1 following treatment with IFN-β, was potentiated in the presence of exogenous α-syn.

These data add to previous evidence indicating endogenous α-syn as a key mediator in ISG induction [1, 2], to document an association between Type-I IFN responses, SNCA expression, and α-syn multimer:monomer dynamics as important determinants of SARS-CoV-2 replication. Again, these results confirm that besides being associated with impaired α-syn multimerization, and reduced α-syn multimer:monomer ratio, SARS-CoV-2 infection alters α-syn levels by acting at transcriptional level as well, with the magnitude of infection likely being directly proportional to SNCA mRNA downregulation. In fact, SARS-CoV-2 synergized with exogenously added α-syn to produce an additional downregulation of SNCA mRNA compared with the untreated infected control. In line with this, infection assays carried out at late-time intervals (3 and 5d) post-infection at 3 different MOIs, showed the presence of a strong, and statistically significant, negative correlation between SARS-CoV-2 replication and SNCA expression within susceptible epithelial lung cell lines (Fig. 6B1, B2). This might represent an attempt of the virus to hamper production of α-syn monomers available for assembly into higher order conformers, which remains to be confirmed by protein analyses at late time post-infection.

In line with these data, preliminary experiments mimicking a more physiological condition of the pulmonary milieu were carried out by setting up a co-culture system of PBMC-epithelial lung cells, as previously described [39]. This model was used to investigate potential associations between SNCA expression in immune cells, and epithelial cell susceptibility to SARS-CoV-2 infection. PBMCs from 8 healthy donors were seeded in the upper chamber on the trans-well, while epithelial cells were cultured in the lower one. In PBMCs, which are refractory to productive SARS-CoV-2 infection, stimulation with the virus led to a two-fold increase in SNCA mRNA compared with Mock cells (Fig. 6C1). Remarkably, SNCA expression in PBMCs was strongly and negatively correlated with SARS-CoV-2 replication in co-cultured CaLu-3 cells, as detected through RT-qPCR (Fig. 6C2). This suggests that SARS-CoV-2-associated increase in SNCA mRNA expression in PBMCs results in the release of soluble mediators that influence the susceptibility of epithelial cells to infection. While further experiments are needed to confirm whether such correlations are reproduced at protein levels, these data highlight a previously unappreciated role of α-syn in SARS-CoV-2 infection within peripheral cells.

## Discussion

As an intrinsically disordered, and highly dynamic protein, alpha-synuclein (α-syn) is widely recognized as a hallmark of neurodegenerative synucleinopathies due to its propensity to misfold and aggregate within nerve cells [19, 40, 41]. The association between neurological sequelae and COVID-19 has widely prompted research focused on the possible role of α-syn in SARS-CoV-2 infection [8–12, 42]. In fact, several studies converge in documenting α-syn accumulation/aggregation following SARS-CoV-2 exposure or infection, or even stimulation/transfection with viral proteins [8–12, 42]. However, the functional significance of α-syn accumulation in SARS-CoV-2 infection remains unclear. In the present study we investigated whether SARS-CoV-2 affects α-syn levels and dynamics within peripheral cells displaying different susceptibility to the live virus. Our results confirm that α-syn is expressed at mRNA level, and occurs constitutively as monomers and multimers within human epithelial lung cells, and umbilical vein endothelial cells (HUVECs). Within CaLu-3 and A549-hACE2 epithelial lung cells, we could detect a decrease in SNCA mRNA expression and/or α-syn immunostaining at late time-intervals (48 h, 3d, 5d) post-SARS-CoV-2 infection. While SNCA-siRNA experiments showed that downregulation of constitutive α-syn marginally affects SARS-CoV-2 replication, administration of IFN-β consistently upregulated α-syn at both mRNA and protein levels, which was associated with suppression of viral replication. Supporting earlier data obtained in West Nile and Venezuelan equine encephalitis infections [2], this suggests that α-syn upregulation occurs as a Type-I IFN-related response coping to hamper viral replication, which is instead antagonized by SARS-CoV-2. Importantly, both immunofluorescence and western blot experiments showed that besides increasing total α-syn levels, IFN-β mostly promotes the formation of multimeric α-syn species, and rescues the multimer:monomer α-syn ratio, which are instead impaired by productive SARS-CoV-2 infection in the presence of excess α-syn monomers. In fact, upon addition, and internalization of α-syn monomers, SARS-CoV-2 lead to a dramatic reduction of α-syn multimers and multimer:monomer ratio compared with Mock treated cells, which was associated with a two-to-three-fold increase of SARS-CoV-2 replication compared with untreated infected cells. This went along with impaired ISG expression and amplification of the pro-inflammatory TNF-A/TNFAR axis, a well-known actor in SARS-CoV-2-induced cytokine storm, which was once again prevented by IFN-β in association with increased amount of α-syn multimers and multimer:monomer ratio. Our data are in line with previous findings supporting a detrimental effect of impaired α-syn multimerization [27, 28], which favors the deposition of insoluble monomers within neuronal cells. Herein, we did not directly address whether insoluble monomer aggregates occur following disruption of α-syn multimer:monomer ratio by SARS-CoV-2 in the presence of exogenous α-syn, as cell-viability assays ruled out any frank cytotoxic effects. This notwithstanding, our data support the hypothesis that impaired α-syn multimerization might play a role in facilitating SARS-CoV-2 replication; conversely, α-syn assembly into multimeric species, and normalization of α-syn multimer:monomer ratio following Type-I IFN might play a role in limiting SARS-CoV-2 replication, at least within peripheral cells. The alterations induced by SARS-CoV-2 in the presence of exogenous α-syn monomers in epithelial cells were not observed in endothelial cells, which are refractory to productive SARS-CoV-2 infection. Rather, within these cells, refractoriness to productive SARS-CoV-2 infection, which was preserved by both exogenous α-syn and IFN-β, was associated with an increase in large α-syn species, as well as α-syn multimer:monomer ratio. Likewise, within human PBMCs which are refractory to productive SARS-CoV-2 infection, stimulation with the virus increased SNCA mRNA expression, which in turn, was negatively correlated with the susceptibility of co-cultured epithelial lung cells to SARS-CoV-2 infection. This suggests that α-syn upregulation in peripheral immune cells, similar to HUVECs that lack productive SARS-CoV-2 replication, might partake in the activation of antiviral, innate immune pathways.

Despite α-syn representing the culprit of synucleinopathies, evolution has preserved this protein to serve a plethora of beneficial functions [19, 41, 43]. In the brain, α-syn accounts for regulating synaptic vesicle dynamics, synapse maintenance, neurotransmitter release, as well as neuronal physiology and plasticity. In fact, α-syn predominantly associates with cell membranes to regulate exocytosis, endocytosis, cellular energy metabolism, and stress responses, which explains why in certain experimental conditions, α-syn expression is critical to prevent or counteract cell damage [44–46]. Such a dichotomy applies also to the oligomerization status of α-syn, as this protein may exist either as a monomer, a variety of aggregation-resistant oligomers/multimers, or as an equilibrium between the two species [24, 28, 47–53]. Notably, α-syn mutants incapable of multimerization were shown to produce in vivo PD-like pathology, pinpointing the key role played by the disruption of the dynamic equilibrium between monomers and multimers as a driver of cellular pathology [27, 28]. α-Syn is also expressed outside the CNS in a variety of tissues and cell populations, including the heart, pancreas, skeletal muscles, gut, kidney, adrenal gland, testis, liver, lung, as well as erythrocytes, endothelial and immune cells [20–25], where it exerts a number of still not fully understood, physiological functions. For instance, the expression of α-syn in tissues such as kidney, liver, and lung, has been related to fetal-to-adult development [23], while its expression in adenocarcinoma lung cells has been correlated with tumor progression [54]. In endothelial cells, SNCA loss-of-function promotes age-related dysfunction going along with hyper-inflammation and elevated blood pressure, which suggests a key role of α-syn in maintaining vascular integrity [55]. In endothelial cells, α-syn also acts as a negative regulator of Weibel-Palade bodies (WPBs) exocytosis [21]. α-Syn also exerts a plethora of immunomodulatory functions, with its upregulation promptly occurring following immune stimulation [3, 17]. Deficiency in T and B lymphocyte development has been documented in SNCA knock‐out mice [56, 57]. Most importantly, α-syn has been shown to promote protective immune reactions against infections. In detail, engineered mice lacking α-syn are characterized by deficient host defense against infectious agents, even in the absence of overt neurological and behavioral alterations [58–60]. α-Syn is required for the development of a normal inflammatory response to bacterial peptidoglycan introduced into the peritoneal cavity, as well as for antigen-specific and T cell responses following intraperitoneal immunization [22].

Finally, α-syn was shown to behave as an ISG and antiviral factor, as documented in West Nile virus infection and Venezuelan equine encephalitis [1, 2]. The authors have proposed two models in which α-syn localizes either: (i) in ER-derived membranes, to modulate virus-induced ER stress signaling, and inhibit viral replication, growth, and injury in the CNS [1]; or (ii) in the nucleus of IFN-β-treated human neurons where it co-localizes with STAT2 to support antiviral, IFN responses [2]. The latter is in line with our results on IFN-β-induced α-syn in the frame of SARS-CoV-2 infection. A protective role of IFN-β against dopamine neuron degeneration associated with α-syn accumulation has been reported in the literature [61]. Intriguingly, the authors documented that such protective effects are associated with a reduction of total but not tetrameric α-syn, suggesting preservation of specific α-syn conformers that are associated with cell protection. Apart from potential cell-specific effects which might explain why IFN-β also increases total α-syn levels in our peripheral cell model, these data support the claim that this cytokine acts by promoting α-syn multimerization towards tetrameric species.

In summary, our data suggest that α-syn accumulation detected both by previous studies and herein following IFN-β administration, might represent a compensatory attempt to cope with SARS-CoV-2-induced antagonism of innate immune responses. This concept is in line with a stream of interpretation suggesting that elevated α-syn expression may serve as a protective factor against RNA viruses, casting the hypothesis that aggregated α-syn contained within neuronal inclusions in PD subjects might be effective in restricting RNA viral replication [3]. Again, supporting its antiviral function, α-syn was shown to promote in vitro formation and stabilization of the SARS-CoV-2 RNA Quadruplex RG-1, a region known to restrict N‐protein translation [16]. Our data argue against the formation of toxic, amyloid α-syn oligomers following SARS-CoV-2 infection, and suggest that α-syn accumulation might rather involve α-syn monomeric species, likely as a consequence of impaired assembly into higher-order conformers, which is also supported by the effects of IFN-β. In fact, in contrast with other studies [8–12, 42], we could not confirm the occurrence of toxic, amyloid species in the presence of either SARS-CoV-2, exogenous α-syn, or IFN-β. This might be related to differences in the experimental models employed. Alternatively, the lack of clear-cut Thioflavin-S-α-syn merging puncta suggests that such α-syn conformations may represent physiological, α-helical multimeric species [24], off-pathway species that do not proceed towards the formation of mature amyloid-like inclusions, or bona fide membrane bound conformers [32]. We wish to underline that while providing indications of the presence of α-syn multimers within our experimental setting, further studies are necessary to provide information about their exact nature, conformation and/or morphology. A missing piece in this puzzle to be addressed in the near future concerns the mechanistic insights into how multimeric α-syn affects SARS-CoV-2 replication on a molecular and cellular level. In this frame, the identification of metabolic and intracellular pathways bridging viral infection, innate immunity and α-syn dynamics will be a key. This might be the case of vesicular trafficking pathways, as well as mitochondrial and lipid dynamics, which are related to both SARS-CoV-2 infection, and α-syn proteostasis.

## Conclusions

**Fig. 7:**
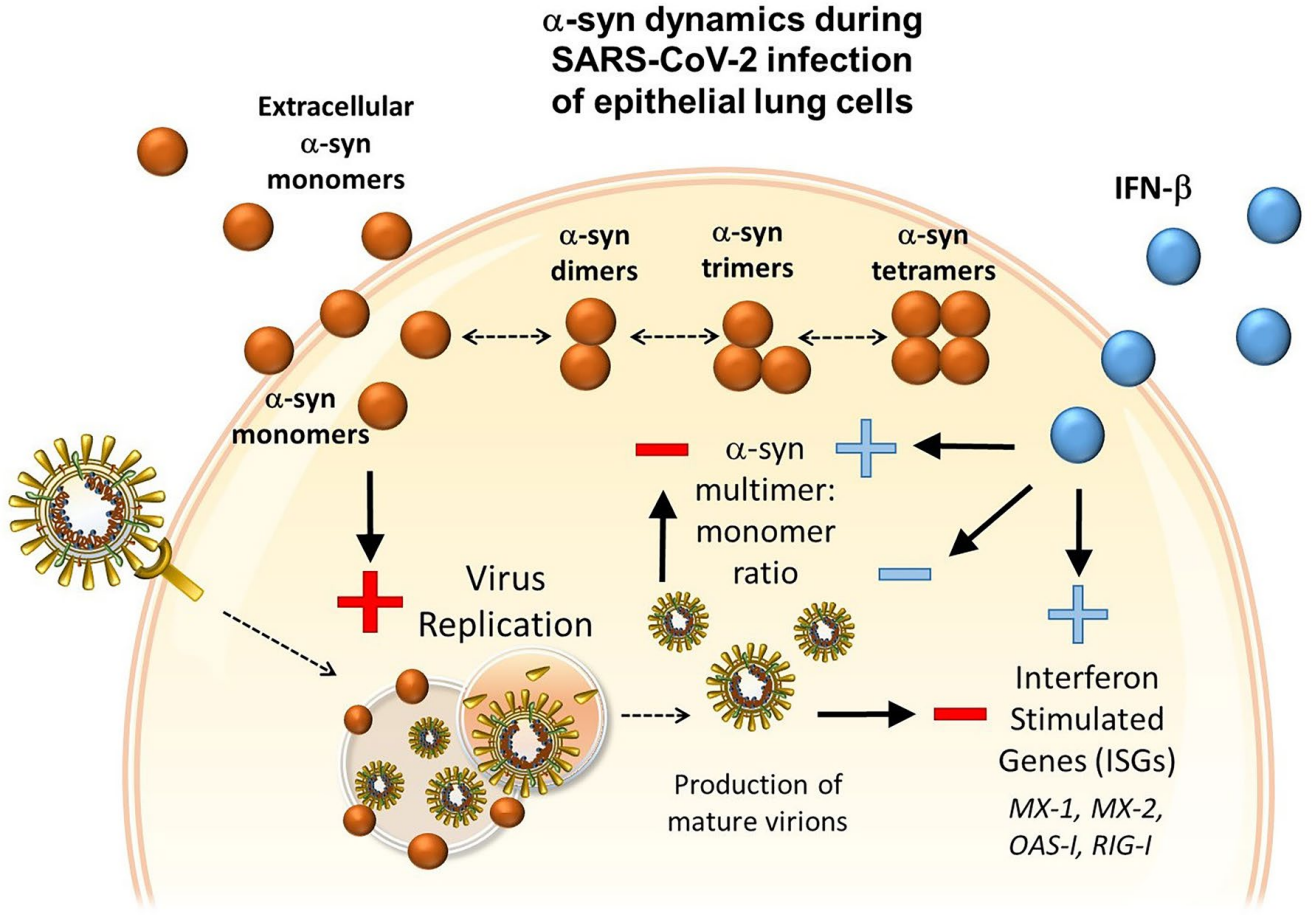
Summary cartoon depicting the opposite effects of extracellular α-syn monomers and IFN-β upon cellular internalization, and related α-syn multimer:monomer dynamics during SARS-CoV-2 infection of susceptible epithelial lung cells

Our data indicate, for the first time, that: (i) α-syn dynamics within peripheral cells are related to the Type-I Interferon (IFN) response against SARS-CoV-2 infection; (ii) productive SARS-CoV-2 infection synergizes with an excess of α-syn monomers to impair α-syn multimerization, which fosters viral replication and downregulation of ISG expression, and (iii) promotion of non-toxic α-syn multimers and upregulation of α-syn multimer:monomer ratio occur as a Type-I IFN-related event which might partake in the cellular defense mechanisms against SARS-CoV-2 replication (Fig. 7). Although recent studies did not detect alterations of α-syn concentration in the sera or CSF of SARS-CoV-2-infected PD patients [62], our data suggests that α-syn levels in peripheral tissues and immune cells should be investigated in the frame of SARS-CoV-2 infection, and potentially, its association with neurological diseases. In vivo studies and future investigations in neuronal cells will be seminal to confirm our findings on the immune-biological role of α-syn, and to elucidate how different α-syn conformers and their dynamics contribute to modulating SARS-CoV-2 infection.

## Materials and methods

### Cell culture, treatments, and SARS-CoV-2 infection

#### Cell cultures

CaLu-3 cells (ATCC, HTB-55^™^, human lung adenocarcinoma) were grown in DMEM supplemented with 10% Fetal Bovine Serum (FBS), 1% non-essential aminoacids (NEAA), and 1% penicillin–streptomycin (PS)/L-glutamine. A549-hACE2 epithelial cells (NR-53522, BEI Resources, NIAID, NIH, human lung adenocarcinoma cells expressing Human Angiotensin-Converting Enzyme 2), and VERO-E6 cells (ATCC, African green monkey epithelial kidney cells) were grown in DMEM supplemented with 10% FBS, and 1% PS/L-glutamine. Human Umbilical Vein Endothelial Cells (HUVECs, Lonza, Walkersville, USA) were grown in EMG-2 medium (Lonza, Walkersville, USA) containing 2% FBS.

#### SARS-CoV-2 infection and treatments

SARS-CoV-2 (strain 2019-nCoV/Italy-INMI1) was expanded in VERO-E6 cells and viral titers were assessed through the TCID_50_ endpoint dilution assay in CaLu-3 and A549-hACE2 epithelial cells. Given the absence of cytopathic effects, viral titers from cell supernatants were determined in order to assess TCID_50_ through quantitative, real-time, Reverse Transcriptase-Polymerase Chain Reaction (RT-qPCR), as previously described [63, 64]. The TCID_50_ values were used to calculate the Multiplicity of Infection (MOI). CaLu-3, and A549-hACE2 cells were seeded in 24-well plates (1.25 × 10^5^, and 0.8 × 10^5^ cells/well, respectively, in a final volume of 500 μL) for 24 h. HUVECs were seeded into a 24-well cell culture plate (0.5 × 10^5^ cells/well), containing 0.5% gelatinized cover slips, and grown to confluence until they reached the cobblestone morphology (7 days).

Type-I IFN (human recombinant IFN-β, 500 IU/ml, BEI resources) and human recombinant α-syn monomers (1 μM, Merck-Sigma), were exogenously pre-administered to cells, both alone and in combination, at 24 h pre-infection. CaLu-3 and A549-hACE2 cells were Mock-infected or infected with SARS-CoV-2 at a MOI of 0.05 for 1 h, while HUVECs were Mock-infected or SARS-CoV-2-infected at a MOI of 1 for 1 h. Cells were washed in PBS to remove unbound virus and cultured in fresh medium. Supernatants and/or cells were collected at different time intervals post-infection (24 and 48 h for CaLu-3, and A549-hACE2; 24, 48, and 72 h for HUVECs), and processed accordingly for different methodological assays.

In a different set of experiments, CaLu-3, and A549-hACE2 cells were seeded at a low density (1.25 × 10^5^, and 0.8 × 10^5^ cells/well) in 12-well plates in a final volume of 1 mL growth medium. Cells were cultured for 24 h and then infected with SARS-CoV-2 at three different MOIs (0.01, 0.005, 0.001) for 1 h. CaLu-3, and A549-hACE2 were washed in PBS, replenished with 1 mL fresh medium, and cultured up to 3 and 5d, respectively. At these time-points, cells were harvested for transcriptional analysis aimed at investigating correlations between the mRNA expression of SNCA and SARS-CoV-2 Nucleocapsid (N) gene.

Eventually, a co-culture system of PBMCs and CaLu-3 was set up as previously described [39]. Briefly, 1.25 × 10^5^ Calu-3 cells were cultured in the lower chamber of a 12 well plate with 0.4 µm pore polycarbonate membrane inserts (Costar, Corning Incorporated, Corning, NY, USA) in 1 mL medium with 10% FBS. The same day, 2 × 10^6^ PBMCs isolated from healthy, SARS-CoV-2-negative volunteers, were cultured in 1 mL RPMI medium with 10% FBS and 15 ng/mL of IL-2 in the upper chamber of the insert membranes placed on the pre-seeded Calu-3 cells. After 24 h, cells were either Mock-infected or challenged with SARS-CoV-2 (2019-nCoV strain 2019-nCoV/Italy-INMI1, Rome, Italy) at a MOI of 0.01 for 1 h. Cells were than thoroughly washed with PBS and refilled with proper growth medium. At 3d post-infection, both PBMCs and Calu-3 cells and supernatants were collected for RNA extraction, and evaluation of SARS-CoV-2 replication rate by using the viral RNA extraction method and the SARS-CoV-2-N mRNA detection protocol described below. Ethical clearance was obtained from the University of Milan Ethics Committee (number 14/22). Written informed consent was obtained after receiving information about use of their biological samples. The biological material was anonymized. All the experiments with SARS-CoV-2 virus were performed in the BSL3 facility.

### Quantification of viral replication from cell supernatants

For assessment of SARS-CoV-2 replication, RNA from cell culture supernatants (volume = 100 μl from 24-well plates; 200 μl from 12-well plates) was extracted through the Maxwell^®^ RSC Instrument (Promega, Fitchburg, WI, USA), and quantified through real time, RT-qPCR by means of well-validated primers for SARS-CoV-2 N gene (2019-nCoV_N2, 2019-nCoV_N2, Forward Primer 5ʹ-TTA CAA ACA TTG GCC GCA AA-3ʹ, Reverse Primer 5ʹ-GCG CGA CAT TCC GAA GAA-3ʹ), as previously described [64]. RT-qPCR was performed through the CFX96 instrument (Bio-Rad). Melting curves besides Cq values were analyzed for primer and reaction specificity. Absolute viral copy number quantification was performed by referring to a standard curve from the quantified 2019-nCoV_N-positive Plasmid Controls (IDT, USA). Before sample analysis outside the BSL3 area, the virus was inactivated according to institutional safety guidelines.

### Cell viability

CaLu-3 and A549-hACE2 cells (3 × 10^4^, and 2 × 10^4^ cells/well, respectively) were seeded in 96-well plates for 24 h and then treated with Type-I IFN (human recombinant IFN-β, 500 IU/ml, BEI resources) and/or human recombinant α-syn monomers (1 μM, S7820, Merk-Sigma). Cells were then either mock-infected or infected with SARS-CoV-2 as detailed above. After 48 h, cell viability was assessed by 3-(4,5-dimethylthiazol-2-yl)-2,5-diphenyltetrazolium bromide (MTT) method. Briefly, 30 μL of MTT (final concentration, 0.5 mg/mL) were added to each well under sterile conditions, and the 96-well plates were incubated for 4 h at 37 °C. Supernatants were removed, and dimethyl sulfoxide (100 µL/well) was added. The plates were then agitated on a plate shaker for 5 min. The absorbance of each well was measured at 490 nm with a Bio-Rad automated EIA analyzer (Bio-Rad Laboratories, Hercules, CA, USA). The viability of Control cells (Mock-infected, vehicle-treated) was considered 100%, while the other conditions were expressed as percentages of control.

For Trypan Blue exclusion assay, cells in 24-well plates were incubated in Cell Dissociation Buffer 1X (Merck-Sigma, Milan, Italy) for 10 min at 37 °C. Then, an equal volume of DMEM was added to the wells to stop the dissociation reaction. Ten μL of cell suspension were mixed and briefly incubated with 10 μL of 0.4% Trypan Blue (Merck-Sigma, Milan, Italy) in 96-well plates. Ten microliters of the mix were loaded on chamber slides and counted with the T20 Automated Cell Counter (Bio-Rad Laboratories, Hercules, CA, USA). Results are expressed as the mean ± SEM from n = 3 independent experiments.

### SNCA RNA interference

To silence SNCA expression, specific small interfering RNA oligonucleotides (SNCA-siRNA, AM16708, Thermo Scientific, Waltham, MA, USA) were used:

Sense 5ʹ-GGGUAUCAAGACUACGAACtt-3ʹ.

Antisense 5ʹ-GUUCGUAGUCUUGAUACCCtt-3ʹ.

A549-hACE2 cells were transfected with 10 nM siRNA, either Non-Targeting negative control (siRNA AM4613 Negative Control No. 2, Thermo Scientific, Waltham, MA, USA) or against SNCA, using Lipofectamine 2000 according to the manufacturer’s instructions (Thermo Scientific, Waltham, MA, USA). Transfected cells were cultured for 24 h before infection with SARS-CoV-2 and harvested/fixed at 48 h post-infection (72 h post-transfection), for cell viability assessment through Trypan Blue exclusion assay, transcriptional analysis through qPCR, or immunofluorescence analysis.

### Immunofluorescence

For immunofluorescence, cells were seeded on Poly-Lysine-coated glass coverslips placed on 24-well plates. At the indicated times post-infection, cells were fixed and permeabilized with different approaches slightly modified from [31] to provide better sensitivity for the detection of total α-syn, monomeric or multimeric α-syn species. In detail, for experiments shown in Figs. 1, 2, and 6, cells were fixed for 15 min in 4% Formaldehyde solution (containing methanol as a stabilizing agent), and then permeabilized with 0.1% Triton 100X for 15 min. This procedure is expected to moderately permeabilize cells allowing rough detection of both α-syn oligomers and monomers. For experiments in Figs. 4, and 5, fixation and permeabilization procedure was modified for better visualization of α-syn monomers vs oligomers/multimers. In detail, for preservation and better visualization of α-syn monomers, cells were fixed in 4% paraformaldehyde (PFA) for 15 min and briefly permeabilized with 0.1% Triton 100X for 10 min. For better visualization of α-syn oligomers/multimers, cells were fixed for 15 min in 4% Formaldehyde solution (containing methanol as a stabilizing agent) and then further permeabilized with 0.3% Triton 100X for 15 min. After thorough washing, cells were incubated in a blocking solution of 5% BSA for 1 h, and then with primary antibodies against α-syn (rabbit mAb, 1:200, BSM-54277R 3H12, Bioss, USA), and SARS-CoV-2 Spike (mouse mAb, 1:1000, 1A9, Genetex, Cat. No. GTX632604, Prodotti Gianni, Milan, Italy) or Nucleocapsid (mouse mAb, 1:1000, E8R1L, #33717, Cell Siganling, Euroclone, Milan, Italy) prepared in 1% BSA for 1 h at RT. Cells were washed thrice in PBS and incubated with Alexa-Fluor-conjugated secondary antibodies raised against the host species of the primary antibodies, namely Goat anti-mouse Alexa Fluor 488 (abcam, ab150113) or 647 (abcam, ab150115), or Goat anti-rabbit Alexa Fluor 488 (abcam, ab150077) or 647 (abcam, ab150079), 1:500 prepared in 1% BSA-PBS. Negative controls were performed by omitting primary antibodies. After 3 × 5 min washes in PBS, coverslips were mounted on Superfrost glass slides using a mounting medium with DAPI (Enzo Life Sciences, Milan, Italy).

### Thioflavin-S-α-syn co-staining

After the incubation with the secondary antibodies, and following 3 × 5 min washes in PBS, CaLu-3 cells were incubated for 15 min at RT in the dark with a solution of 0.05% w/v Thioflavin-S (T1892, Merck-Sigma, Milan, Italy), which was freshly prepared in 50% ethanol/water and 0.22 μm filtered. Cells were washed twice with 50% ethanol for 10 min each, and then washed once with 80% ethanol for 20 min. Eventually, cells were washed in PBS, briefly rinsed with water, and coverslips were mounted on Superfrost glass slides using a mounting medium with DAPI (Enzo Life Sciences, Milan, Italy). Confocal images were acquired on a TCS SP8 System equipped with a DMi8 inverted microscope and a HC PL APO 40 × /1.30 Oil CS2 (Leica Microsystems, Wetzlar, Germany) at a resolution of 1024 × 1024 pixels.

### Quantification of SARS-CoV-2 Nucleocapsid (N)-positive cells

SARS-CoV-2-infected cells were quantified by counting N-positive cells/total cells per microscopy field by using Image J software (NIH, Bethesda, MD, USA). Counts per performed from at least n = 3 microscopy fields per experimental group, and per each independent experiment. At least 300 total cells were counted per each experimental group. Values were expressed as percent N-positive cells ± SD.

### RNA extraction and transcriptional analyses through real-time qPCR

For transcriptional analyses, cells were collected in collected in RNAzol^®^ (TEL-TEST Inc., Friendswood, TX, USA) and RNA extraction was performed through the phenol–chloroform method, as previously described [63]. RNA was quantified by the Nanodrop 2000 Instrument (Thermo Scientific, Waltham, MA, USA). One μg of RNA was purified from genomic DNA with RNase-free DNase (RQ1 DNase; Promega) and reverse transcribed into cDNA with Moloney murine leukemia virus reverse transcriptase along with random hexanucleotide primers, oligo dT, and dNTPs (Promega, Fitchburg, WI, USA). cDNA (25 ng) was amplified and quantified by real-time qPCR (CFX96 connect, Bio-Rad, Hercules, CA, USA) through SYBR Green PCR mix (Promega, Fitchburg, WI, USA). Negative controls (distilled water), as well as positive controls (human cDNA), were included in each run. Results for gene expression analyses were calculated by the 2^−ΔΔCt^ equation. Melting curves besides Cq values were analyzed for primer and reaction specificity. Results are presented as the percent mean ± SEM of the relative expression units to an internal reference sample, and normalized to the Glyceraldehyde-3-Phosphate Dehydrogenase** (**GAPDH) housekeeping gene. Results show the quantifications from at least n = 3 independent experiments. The following genes were analyzed: synuclein alpha (SNCA), Interferon beta (IFNB), SARS-CoV-2 Nucleocapsid (N2), Signal transducer and activator of transcription 1 (STAT1), Myxovirus Resistance Protein 1 (MX1), Myxovirus Resistance Protein 2 (MX2), 2'-5'-oligoadenylate synthetase 1 (OAS1), retinoic acid-inducible gene I (RIG-I), tumor necrosis factor alpha (TNFA), tumor necrosis factor receptor superfamily 1A (TNFRSF1A), Toll-like receptor 8 (TLR8), Toll-like receptor 9 (TLR9). Primers for STAT1, and TNFRSF1A were purchased as already optimized (PrimePCR, Bio-Rad). The sequences of the remaining primers are listed in Additional file 1: Table S1.

### Western blot

Cells were collected in ice-cold lysis buffer (RIPA buffer with 1% Triton X-100, and 0.1% SDS) supplemented with a cocktail of protease and phosphatase inhibitors (cOmplete and PhosSTOP; Roche Applied Science, Mannheim, Germany). Samples were incubated on ice for 30 min on a platform rotator, sonicated, and then centrifuged at maximum speed for 30 min at 4 °C. Protein concentration was determined through BCA protein assay kit (Pierce, USA). 50 μg proteins per sample were prepared by combining the appropriate volume with 5 × Laemmli SDS sample buffer (final 1 ×) and Milli-Q H_2_O. Samples were boiled for 5 min and loaded on 4–12% SDS-PAGE gel (Bio-Rad), which was run at 150 V for 1–1.5 h. Proteins were transferred on PVDF membrane through The Trans-Blot Turbo Transfer System TM and Transfer packTM (Bio-Rad). To preserve α-syn protein, membrane fixation with 0.4% PFA for 30 min at RT was performed [31]. Prior to the blocking step, the membrane was imaged as a loading control for normalization to total protein. The membrane was blocked in 1 × TBS with 5% non-fat milk for 1 h at RT, washed in TBS-0.2% Tween, and incubated overnight at 4 °C with rabbit anti- α-syn antibody (1:800, BSM-54277R 3H12, Bioss, USA) in blocking buffer with 2.5% non-fat dry milk. The day after, the blot was washed 3 × 5 min with 1 × TBS-0.2% Tween and incubated for 1 h with HRP-conjugated goat anti-rabbit secondary antibody (1:5000, STAR208P, Bio-Rad) in blocking buffer with 2.5% non-fat dry milk. The blot was washed 3 × 5 min with 1 × TBS-0.2% Tween and then incubated for 5 min with Clarity Western ECL substrate and visualized with a ChemiDoc MP imaging system (Bio-Rad). Results were analyzed using the Image Lab software (Bio-Rad). For each experiment, a representative blot is shown, and the graphs show to the mean ± SEM of raw normalized values obtained from indicated (n) independent experiments.

### Statistical analysis

The GraphPad Prism software package (GraphPad Software, San Diego, CA, USA) was used to generate all the graphs. Data normality was assessed through the Shapiro–Wilk test. In case of normal distribution, the statistical significance was evaluated using the unpaired Student’s *t test* (single comparisons), or one-way, or two-way ANOVA (as appropriate, for multiple comparisons), followed by multiple testing correction by false discovery rate (FDR) through the Two-stage linear step-up procedure of Benjamini, Krieger and Yekutieli. When data normality was non confirmed, non-parametric Wilcoxon ranked or Kruskal Wallis tests were applied. Obtained q values corresponding to adjusted p values were shown in the graphs with statistical significance set as *p < 0.05, **p < 0.01, ***p < 0.001, ****p < 0.0001. Pearson’s r coefficient was calculated for correlation analyses followed by two-way, paired Student’s *t test*. To avoid graphs overcrowding, p values were shown for statistically significant groups of interest only. Results are expressed as mean ± SEM or SD of the indicated *n* values, as specified in the figure legends.

### Supplementary Information

**Additional file 1: Figure S1.** A. Trypan Blue Exclusion assay in A549-hACE2 cells. Transfection with either NT-siRNA or SNCA-siRNA does not significantly affect cell viability compared with Mock- or SARS-CoV-2-infected CTR cells. Again, transfection with SNCA-siRNA does not significantly affect cell viability compared with NT-siRNA in either Mock- or SARS-CoV-2-infected cells. Results are expressed as mean ± SEM from n = 3 independent experiments. Statistical analysis was performed by applying Two-way ANOVA. ns = non-significant. B and C. MTT assay rules out any cytotoxic effects of exogenous α-syn, IFN-β, or their combination, in A549-hACE2 (B) and CaLu-3 cells (C). Results are expressed as mean ± SEM from n = 3 independent experiments. Statistical analysis was performed through Wilcoxon matched-pairs signed rank test (to test for differences between MOCK and VIRUS groups) and the Kruskal–Wallis test (to test for differences among various treatments within the same group). ns = non-significant. **Figure S2.** Thioflavin-S-α-syn co-staining rules out an amyloid nature of permeabilization-resistant α-syn species induced by IFN-β. Representative immunofluorescence images for α-syn protein combined with Thioflavin-S (Th-S) staining in Mock- and SARS-CoV-2-Infected CaLu-3 epithelial lung cells 48 h post-infection (MOI 0.05), in the absence or presence of exogenous α-syn monomers, or IFN-β. Cells were fixed for 15 min in Formaldehyde solution, followed by 15 min permeabilization with 0.3% Triton X-100. Bars correspond to 20 μm. **Figure S3.** A. HUVECs immunofluorescence negative controls. Negative control was performed in Mock cells by omitting primary antibodies. Some non-specific background signal for the secondary antibody Goat anti-mouse Alexa Fluor 488 (ab150113) was observed in HUVECs, likely due to their inherent fluorescence in the green spectrum. B. Trypan Blue Exclusion Assay in HUVECs. Exogenous α-syn, or IFN-β did not produce cytotoxicity in HUVECs. Results are shown as mean ± SEM from n = 3 independent experiments. Data were analyzed by applying Two-Way ANOVA. ns = non-significant. **Figure S4.** Representative Western Blot at low exposure showing distribution of exogenously added α-syn, as well as SARS-CoV-2 N protein immunostaining in A549-hACE2 cells at 48 h post infection, in the absence and presence of IFN-β. Endogenous α-syn was not detected at short exposure. Exogenous α-syn addition enhanced SARS-CoV-2 N protein immunostaining compared with untreated infected cells, which disappeared following combination with IFN-β. **Figure S5.** In the absence of productive SARS-CoV-2 infection, exogenous α-syn administration fails to decrease α-syn multimers and multimer:monomer ratio. A. Western blot for α-syn detection in HUVECs 78 h post- Mock and SARS-CoV-2 infection in the absence and presence of IFN-β or exogenous α-syn monomers. Blots of the experimental groups without exogenous α-syn are only representative, while groups treated with exogenous α-syn were quantified and graphed (B and C). Normalization was performed against total protein (Loading control). Results show raw normalized values presented as mean ± SEM from n = 2 independent experiments. Multimer:monomer α-syn ratio is expressed as absolute value calculated as “multimers/monomers”. Data were analyzed by applying Two-Way ANOVA (for total, multimer, monomer α-syn quantification) or One Way ANOVA (for multimer:monomer ratio). ns = non-significant. **Table S1.** Sequences of the primers employed in the study. FW forward; RV reverse. Glyceraldehyde-3-Phosphate Dehydrogenase (GAPDH), synuclein alpha (SNCA), SARS-CoV-2 Nucleocapsid 2 (SARS-CoV-2 N2); Interferon beta (IFNB), tumor necrosis factor alpha (TNFA), 2'-5'-oligoadenylate synthetase 1 (OAS1), retinoic acid-inducible gene I (RIG-I), Myxovirus Resistance Protein 1 (MX1), Myxovirus Resistance Protein 2 (MX2), Toll-like receptor 8 (TLR8), Toll-like receptor 9 (TLR9).

## Data Availability

Data generated during this study are included in the present manuscript and its supplementary information files. Raw data are available from the corresponding author upon request.
