# Supplementary material for: Alpha-synuclein dynamics bridge Type-I Interferon response and SARS-CoV-2 replication in peripheral cells
Source: Biol Res. 2024 Jan 9;57:2. doi: 10.1186/s40659-023-00482-x (PMC10775536; doi:10.1186/s40659-023-00482-x)
Supplement: Supplementary file 1 — Additional file 1: Figure S1. A. Trypan Blue Exclusion assay in A549-hACE2 cells. Transfection with either NT-siRNA or SNCA-siRNA does not significantly affect cell viability compared with Mock- or SARS-CoV-2-infected CTR cells. Again, transfection with SNCA-siRNA does not significantly affect cell viability compared with NT-siRNA in either Mock- or SARS-CoV-2-infected cells. Results are expressed as mean ± SEM from n = 3 independent experiments. Statistical analysis was performed by applying Two-way ANOVA. ns = non-significant. B and C. MTT assay rules out any cytotoxic effects of exogenous α-syn, IFN-β, or their combination, in A549-hACE2 (B) and CaLu-3 cells (C). Results are expressed as mean ± SEM from n = 3 independent experiments. Statistical analysis was performed through Wilcoxon matched-pairs signed rank test (to test for differences between MOCK and VIRUS groups) and the Kruskal–Wallis test (to test for differences among various treatments within the same group). ns = non-significant. Figure S2. Thioflavin-S-α-syn co-staining rules out an amyloid nature of permeabilization-resistant α-syn species induced by IFN-β. Representative immunofluorescence images for α-syn protein combined with Thioflavin-S (Th-S) staining in Mock- and SARS-CoV-2-Infected CaLu-3 epithelial lung cells 48 h post-infection (MOI 0.05), in the absence or presence of exogenous α-syn monomers, or IFN-β. Cells were fixed for 15 min in Formaldehyde solution, followed by 15 min permeabilization with 0.3% Triton X-100. Bars correspond to 20 μm. Figure S3. A. HUVECs immunofluorescence negative controls. Negative control was performed in Mock cells by omitting primary antibodies. Some non-specific background signal for the secondary antibody Goat anti-mouse Alexa Fluor 488 (ab150113) was observed in HUVECs, likely due to their inherent fluorescence in the green spectrum. B. Trypan Blue Exclusion Assay in HUVECs. Exogenous α-syn, or IFN-β did not produce cytotoxicity in HUVECs. Results are shown [file 40659_2023_482_MOESM1_ESM.pdf]

Additional file 1

Alpha-synuclein dynamics bridge Type-I Interferon response and SARS-CoV-2 replication in peripheral cells.

Fiona Limanaqi[1*], Silvia Zecchini[1], Irma Saulle[1,2], Sergio Strizzi[1], Claudia Vanetti[1], Micaela Garziano[1,2], Gioia Cappelletti[1], Debora Parolin[1], Sonia Caccia[1], Daria Trabattoni[1], Claudio Fenizia[1,2], Mario Clerici[2,3], Mara Biasin[1*]

1. Department of Biomedical and Clinical Sciences, University of Milan, Via G.B. Grassi, Milan, Italy.

2. Department of Pathophysiology and Transplantation, University of Milan, Via Francesco Sforza, Milan, Italy.

3. IRCCS Fondazione Don Carlo Gnocchi, 20148 Milan, Italy.

*Correspondence: fiona.limanaqi@unimi.it; mara.biasin@unimi.it

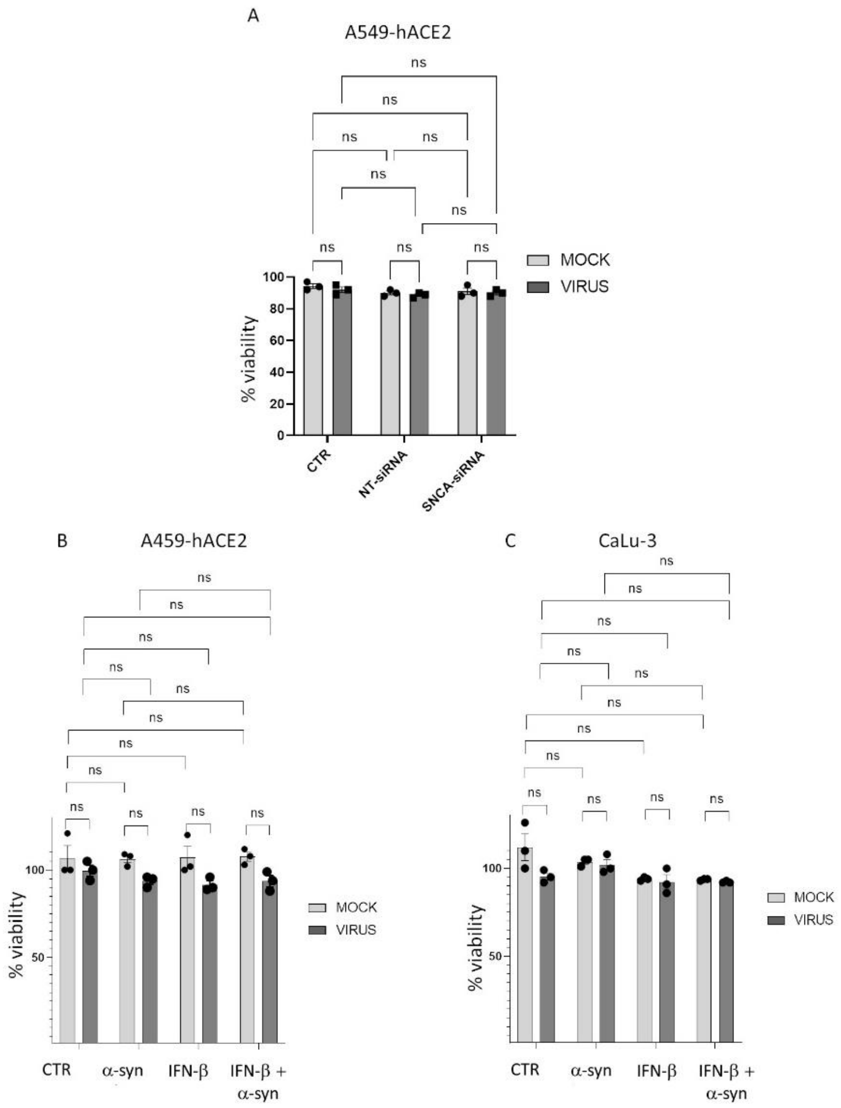

Additional file 1: Fig. S1. A. Trypan Blue Exclusion assay in A549-hACE2 cells. Transfection with either NT-siRNA or SNCA-siRNA does not significantly affect cell viability compared with Mock- or SARS-CoV-2-infected CTR cells. Again, transfection with SNCA-siRNA does not significantly affect cell viability compared with NT-siRNA in either Mock- or SARS-CoV-2-infected cells. Results are expressed as mean ± SEM from n=3 independent experiments. Statistical analysis was performed by applying Two-way ANOVA. ns=non-significant. **B and C.** MTT assay rules out any cytotoxic effects of exogenous α-syn, IFN-β, or their combination, in A549-hACE2 (**B**) and CaLu-3 cells (**C**). Results are expressed as mean ± SEM from n=3 independent experiments. Statistical analysis was performed through Wilcoxon matched-pairs signed rank test (to test for differences between MOCK and VIRUS groups) and the Kruskal-Wallis test (to test for differences among various treatments within the same group). ns=non-significant.

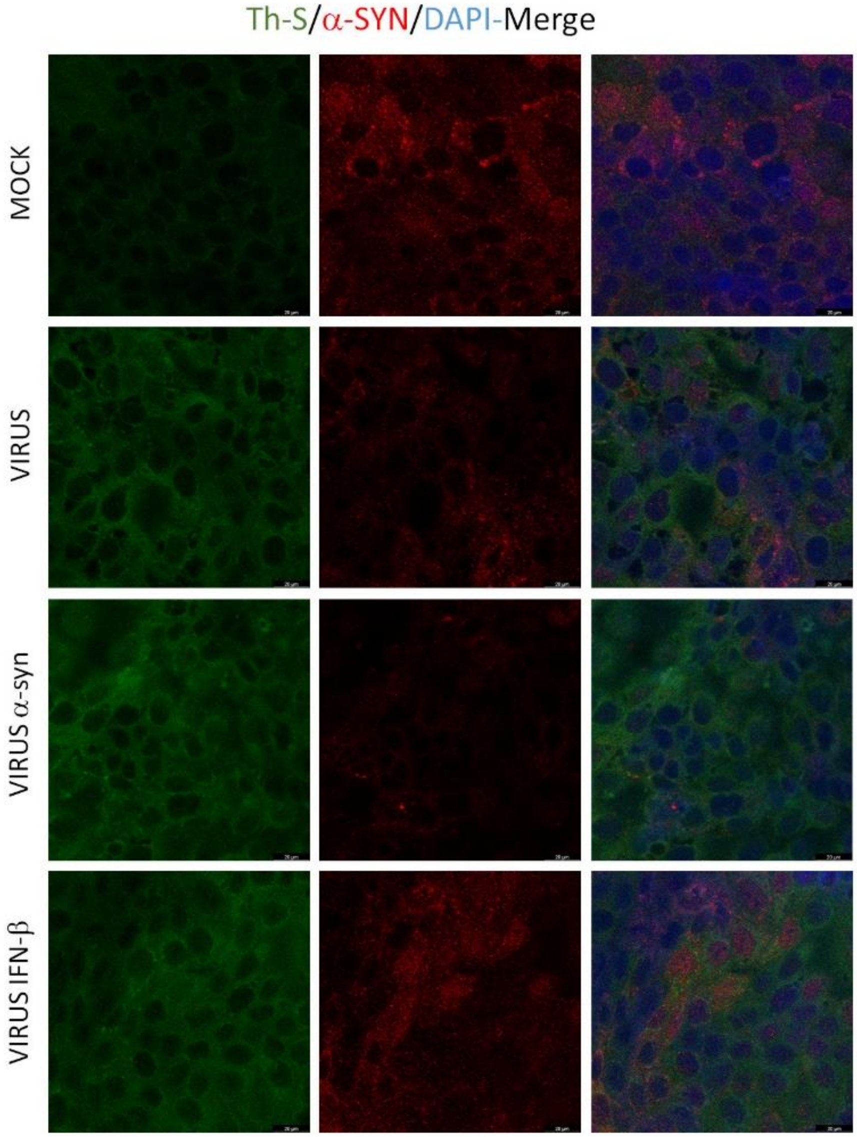

Additional file1: Fig. S2. Thioflavin-S-α-syn co-staining rules out an amyloid nature of permeabilization-resistant α-syn species induced by IFN-β. Representative immunofluorescence images for α-syn protein combined with Thioflavin-S (Th-S) staining in Mock- and SARS-CoV-2-Infected CaLu-3 epithelial lung cells 48h post-infection (MOI 0.05), in the absence or presence of exogenous α-syn monomers, or IFN-β. Cells were fixed for 15min in Formaldehyde solution, followed by 15min permeabilization with 0.3% Triton X-100. Bars correspond to 20 μm.

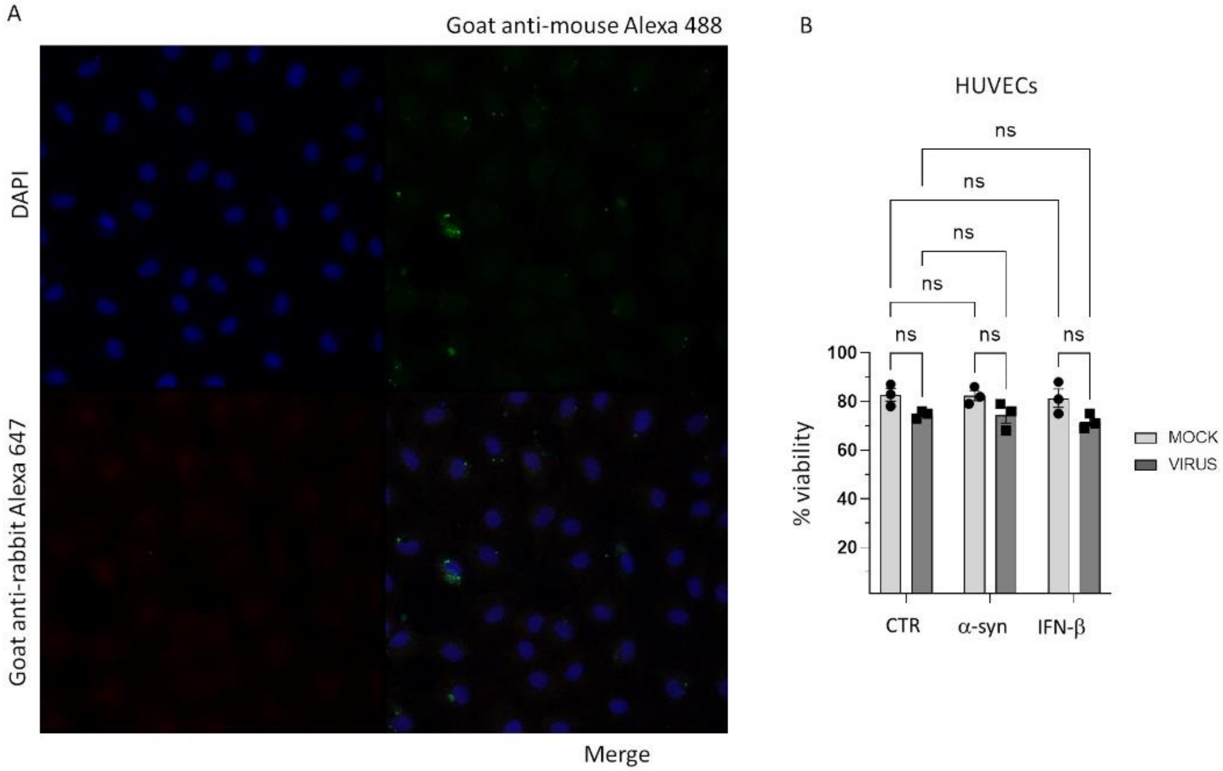

Additional file 1: Fig. S3. A. HUVECs immunofluorescence negative controls**.** Negative control was performed in Mock cells by omitting primary antibodies. Some non-specific background signal for the secondary antibody Goat anti-mouse Alexa Fluor 488 (ab150113) was observed in HUVECs, likely due to their inherent fluorescence in the green spectrum. **B.** Trypan Blue Exclusion Assay in HUVECs. Exogenous α-syn, or IFN-β did not produce cytotoxicity in HUVECs. Results are shown as mean ± SEM from n=3 independent experiments. Data were analyzed by applying Two-Way ANOVA. ns=non-significant

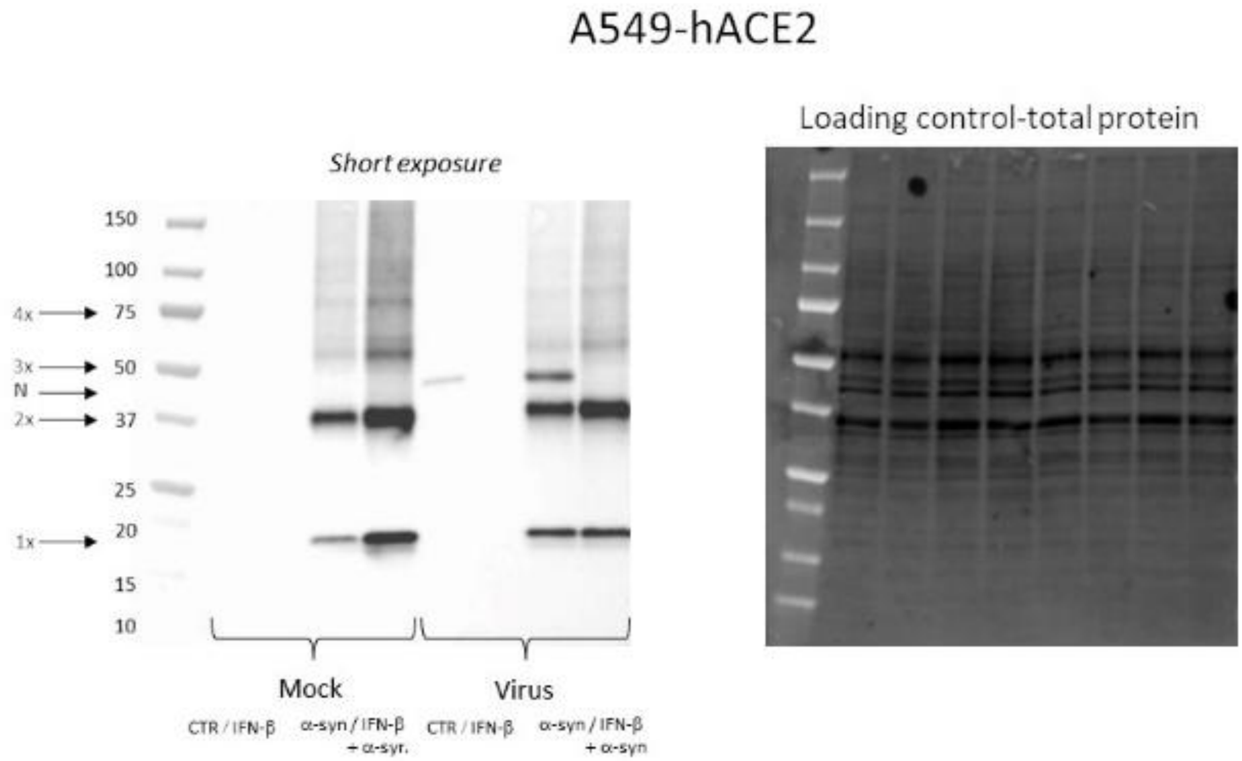

Additional file 1: Fig. S4. Representative Western Blot at low exposure showing distribution of exogenously added α-syn, as well as SARS-CoV-2 N protein immunostaining in A549-hACE2 cells at 48h post infection, in the absence and presence of IFN-β. Endogenous α-syn was not detected at short exposure (Mock, CTR, IFN-β). Exogenous α-syn addition enhanced SARS-CoV-2 N protein immunostaining compared with untreated infected cells, which disappeared following combination with IFN-β.

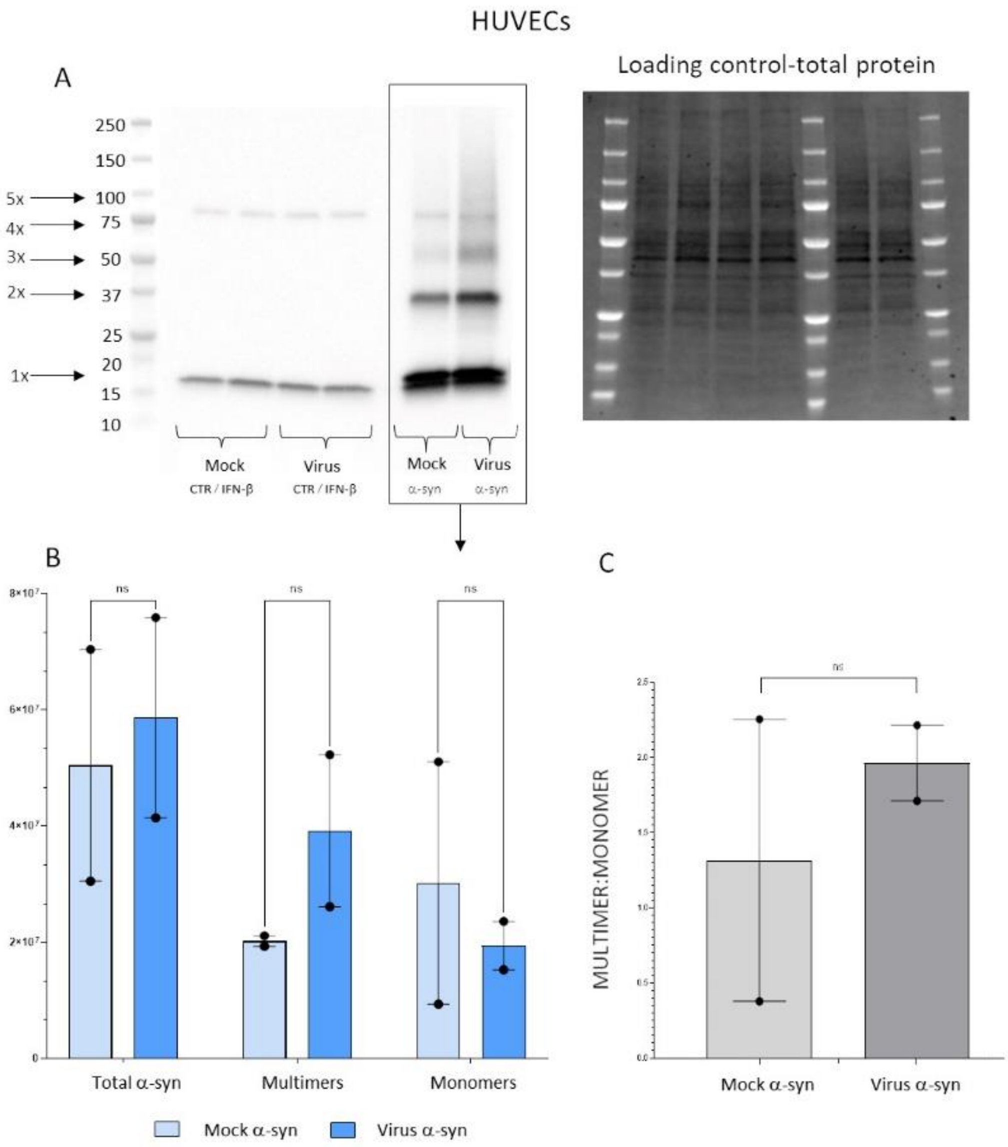

Additional file 1: Fig. S5. In the absence of productive SARS-CoV-2 infection, exogenous α-syn administration fails to decrease α-syn multimers and multimer:monomer ratio. **A.** Western blot for α-syn detection in HUVECs 78h post- Mock and SARS-CoV-2 infection in the absence and presence of IFN-β or exogenous α-syn monomers. Blots of the experimental groups without exogenous α-syn are only representative, while groups treated with exogenous α-syn were quantified and graphed (**B and C**). Normalization was performed against total protein (Loading control). Results show raw normalized values presented as mean ± SEM from n=2 independent experiments. Multimer:monomer α-syn ratio is expressed as absolute value calculated as "multimers/monomers". Data were analyzed by applying Two-Way ANOVA (for total, multimer, monomer α-syn quantification) or One Way ANOVA (for multimer:monomer ratio). ns=non-significant.

Gene	FW 5′-3′	RV 5′-3′
GAPDH	CGGATTTGGTCGTATTGGG	GCTTCCCGTTCTCAGCCTTG
SNCA	GAAGGAGCCCCACAGGAAGGA	TGGGCACATTGGAACTGAGCA
SARS-CoV-2 N2	TTACAAACATTGGCCGCAAA	GCGCGACATTCCGAAGAA
IFNB	GACGCCGCATTGACCATCTA	GACATTAGCCAGGAGGTTCTCA
TNFA	TCTTCTCGAACCCCGAGTGA	CCTCTGATGGCACCACCAG
OAS1	TCAGAAATACCCCAGCCAAATCTC	TGAGGAGCCACCCTTTACCA
RIGI	GGCTTCGCCACACCAAGA	CGCTCCCGTTTCACCTCTG
MX1	CCAGAGGCAGGAGACAATCAGC	TCTTCAGGTGGAACACGAGGTT
MX2	GTGGAGCCCGCCCTTAGC	TCTGCCTTTGCTGTGTGTTTCA
TLR8	AGGCTACAGGTCTCTTTCCA	GCTCATTTATCACCCAGTCA
TLR9	GCATCTCGCAGGCAGTCAAT	TGCTCGTGGTAGAGGTCCAG

Additional file 1: Table 1. Sequences of the primers employed in the study. FW forward; RV reverse. Glyceraldehyde-3-Phosphate Dehydrogenase (GAPDH), synuclein alpha (SNCA), SARS-CoV-2 Nucleocapsid 2 (SARS-CoV-2 N2); Interferon beta (IFNB), tumor necrosis factor alpha (TNFA), 2'-5'-oligoadenylate synthetase 1 (OAS1), retinoic acid-inducible gene I (RIG-I), Myxovirus Resistance Protein 1 (MX1), Myxovirus Resistance Protein 2 (MX2), Toll-like receptor 8 (TLR8), Toll-like receptor 9 (TLR9).
